# Artificial neural network for cytocompatibility and antibacterial enhancement induced by femtosecond laser micro/nano structures

**DOI:** 10.1186/s12951-022-01578-4

**Published:** 2022-08-06

**Authors:** Libin Lu, Jiaru Zhang, Kai Guan, Jin Zhou, Fusong Yuan, Yingchun Guan

**Affiliations:** 1 Advanced Manufacturing Center, Ningbo Institute of Technology, Beihang University, Ningbo, 315100 China; 2 School of Mechanical Engineering & Automation, Beihang University, Beijing, 100083 China; 3 Department of Radiotherapy, The Third Hospital of Zhangzhou City, Zhangzhou, 363005 Fujian China; 4 Key Laboratory for Biomechanics and Mechanobiology of Ministry of Education, School of Biological Science and Medical Engineering, Beihang University, Beijing, 100083 China; 5 National Center of Stomatology & National Clinical Research Center for Oral Diseases, Beijing, 100081 China; 6 National Engineering Laboratory of Additive Manufacturing for Large Metallic Components, Beihang University, 37 Xueyuan Road, Beijing, 100083 China

**Keywords:** Femtosecond laser microprocessing, Hierarchical micro/nano-structure, Antibacterial activity, Cytocompatibility, GA-BP neural network

## Abstract

The failure of orthopedic and dental implants is mainly caused by biomaterial-associated infections and poor osseointegration. Surface modification of biomedical materials plays a significant role in enhancing osseointegration and anti-bacterial infection. In this work, a non-linear relationship between the micro/nano surface structures and the femtosecond laser processing parameters was successfully established based on an artificial neural network. Then a controllable functional surface with silver nanoparticles (AgNPs) to was produced to improve the cytocompatibility and antibacterial properties of biomedical titanium alloy. The surface topography, wettability, and Ag^+^ release were carefully investigated. The effects of these characteristics on antibacterial activity and cytocompatibilty were also evaluated. Results show that the prepared surface is hydrophobic, which can prevent the burst release of Ag^+^ in the initial stage. The prepared surface also shows both good cytocompatibility toward the murine calvarial preosteoblasts MC3T3-E1 cells (derived from Mus musculus (mouse) calvaria) and good antibacterial effects against Gram-negative (*E. coli*) and Gram-positive (*S. aureus*) bacteria, which is caused by the combined effect of appropriate micro/nano-structured feature and reasonable Ag^+^ release rate. We do not only clarify the antibacterial mechanism but also demonstrate the possibility of balancing the antibacterial and osteointegration-promoting properties by micro/nano-structures. The reported method offers an effective strategy for the patterned surface modification of implants.

**Graphical Abstract:**

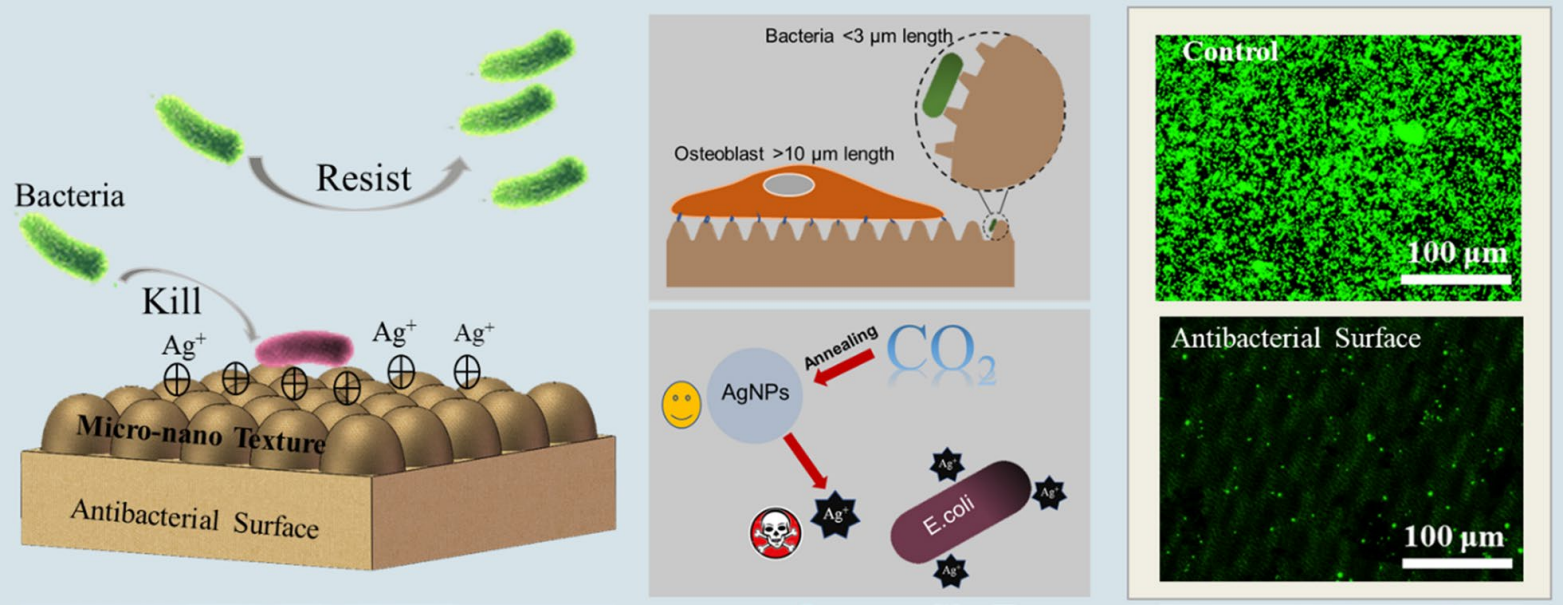

**Supplementary Information:**

The online version contains supplementary material available at 10.1186/s12951-022-01578-4.

## Background

Poor osseointegration and biomaterial-associated infections are the main causes of the failure of orthopedic and dental implants [1]. The implant surface/interface properties can indirectly affect the protein adsorption in the initial stage, and subsequently, affect the adhesion and proliferation behaviour of cell/bacterial [2]. Various surface modification methods such as inorganic coating [3], organic coating[4] have been used to modify the implant surface. Though the surface bioactivity can be improved, the coating thickness is difficult to control and the adhesion strength of the coating is poor, which cannot completely satisfy the clinical requirements.

It has been proved that the surface topology of an implant can affect the cell response through contact guidance, which will change the surface biological properties of the implant, resulting in improved biocompatibility [5]. So, unceasing efforts have been made to use topological structures to modify the implant surface. Rene et al. [6] used sandblasted acid-etched method to modify the pure Ti surface, and the results showed that the surface microstructure and surface energy could induce mesenchymal stem cells to osteoblast lineage. Barbara et al. [7] prepared rectangular grooves (width and height: 2 µm) and cubic pillars (pillar dimensions: 2 × 2 × 5 µm and 5 × 5 × 5 µm) on silicon wafers by a deep reactive-ion etching method, then the prepared samples were sputter-coated with 100 nm titanium. Results indicated that osteoblasts show significantly lower proliferative rates of 66.6% on the pillared surfaces, in comparison to the planar control surface (79.2%), while on the micro-grooved surface, the cell proliferation rate was 78.6%, which was similar to that of on the planar control surface. Li et al. [8] presented a new strategy of surface modification on the Ti-19Zr-10Nb-1Fe (TZNF) alloy by nano-HA and TiO_2_ nanotubes composite coating. TiO_2_ nanotubes with 87 ± 21 nm were successfully formed on TZNF alloy, and nano-HA coating was synthesized on the TZNF-NTs by electrochemical deposition. They found that the nanotube and nano-HA surfaces could improve the proliferation and adhesion of osteoblasts, and cell proliferation rate on the TZNF-NTs/HA surface was 30% higher than that of on the TZNF surface.

Bacteriostasis is another significant property for the implant, so many studies have also explored the surface structures with antibacterial properties [9–11]. Inspired by the antimicrobial attachment performance of lotus leaf, Cheng et al. [12] investigated the micro/nano-scale roughness effects on surface wetting by intentionally altering the lotus leave structures while keeping the chemical composition approximately the same, results showed that the combination of the microscale mounds and the nano-scale hair-like structures caused the anti-adhesion and self-cleaning ability. Bhadra et al. [13] fabricated nanoarrays on titanium surfaces to mimic the surface architecture of dragonfly wings using hydrothermal etching process, these fabricated titanium surfaces showed selective antimicrobial activities to *P. aeruginosa* (50%) and *S. aureus* (20%).

The topological structure modification for implants has been developed greatly in recent years, and lots of studies have been conducted to produce biocompatibility or antibacterial surface on implants [14]. The implants need to have both good biocompatibility and antibacterial property in clinical applications. Unfortunately, a single scale of topological structures is difficult to improve the biocompatibility and antibacterial property at the same time. Moreover, the optimal topological structure is usually obtained through a very complex procedure and can not be precisely controlled according to the different sizes of cells or bacteria. Therefore, it is necessary to prepare a controllable topological structure that is both beneficial to cell growth and bacteriostasis.

In this work, a GA-BP neural network was successfully established for predicting and controlling the laser-induced surface structures. Three different hierarchical micro/nano-structures were designed to investigate the topological structures with simultaneous biocompatibility and antibacterial property. MC3T3-1 cells were used to investigate the cytocompatibility of the prepared surface. Two kinds of bacteria commonly associated with infections *E. coli* (Gram-negative bacteria) and *S. aureus* (Gram-positive bacteria) were used for antibacterial study in vitro.

## Methods

### Materials and sample preparation

Medicinal Ti alloy (Ti6Al4V) disks with diameter of 10 mm and thickness of 2 mm are used in this work. Each sample is mechanically polished with silicon carbide paper (No. 600–4000) and ultrasonically cleaned in ethanol for 10 min. The hierarchical micro/nano-structures covering AgNPs are fabricated in three steps. The first step is the fabrication of hierarchical micro/nano-structures by femtosecond laser ablation on Ti alloy substrates. These hierarchical micro/nano-structures serve as the mechanical skeletons for the subsequently formed AgNPs. The used femtosecond laser is a Yb:KGW solid-state laser system (Pharos, Light Conversion, Lithuania) with a central wavelength of 1030 nm and pulse duration of 230 fs. The laser beam polarization is horizontal and all the samples were processed in focus with the focal plane of 162 mm, while the spot size of around 35 μm. The hierarchical micro/nano-structures are related to the laser-processing parameters and can be modulated precisely over a wide range. Experiments are carried out at normal incidence in the air atmosphere. After laser texturing, the surfaces are ultrasonically cleaned by ethanol. The second step is depositing Ag film with 20 nm thick on the textured surface using a high vacuum metal coating machine system (Discovery 635, Denton, USA). The third step is annealing the substrate in a vacuum furnace (DZF-6021, Yiheng, China) at 200 °C for 1 h. The surface characterization was carried out after the prepared samples were exposed to ambient air for 24 h for sufficient decomposition of carbon dioxide.

### Surface characterization

The surface topography of the samples is observed using a field emission scanning electron microscope (FE-SEM, Quanta 450 FEG, FEI, USA) and an atomic force microscope (AFM, ICON, Bruker, Germany). The surface chemistry composition is characterized by X-ray photoelectron spectroscopy (XPS, ESCALAB 250XI, Thermo Fisher Scientific, USA). The line shape of Tougaard is chosen to analyze the XPS spectrums through the Avantage (Ver 5.976, Thermo Fisher Scientific, USA). The wettability of the surface is measured by an OCA15EC system (DataPhysics, Germany) under atmospheric conditions. The static water contact angles (WCA) are measured by the auto-pipette system with 5 μL of water deposited on the top sample surface at room temperature.

### Design of GA-BP neural network model

**Fig.1:**
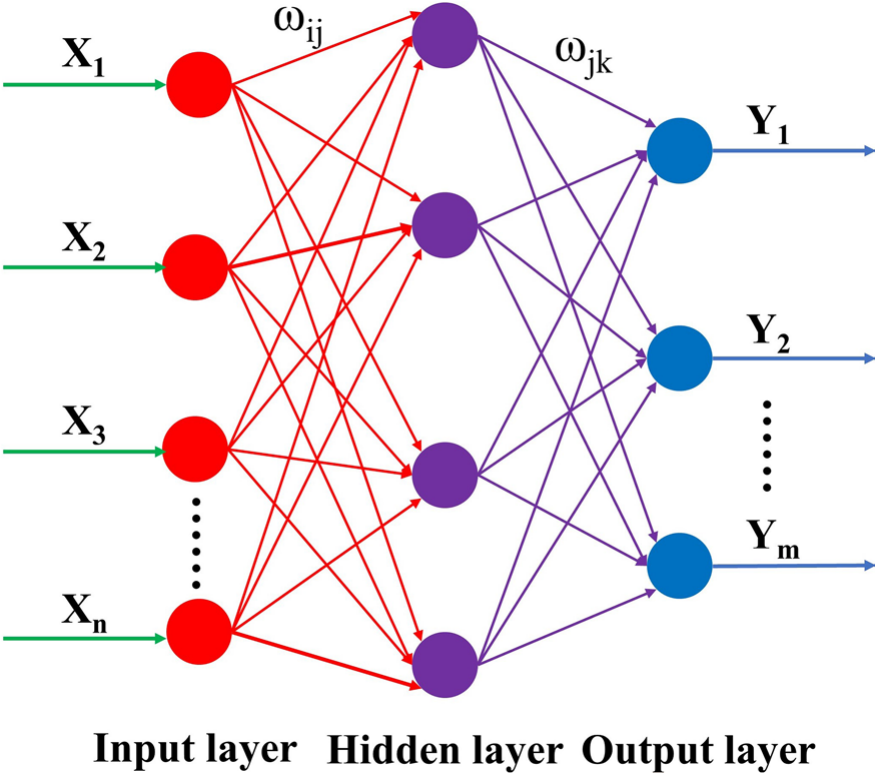
Typical BP neural network model

**Fig.2:**
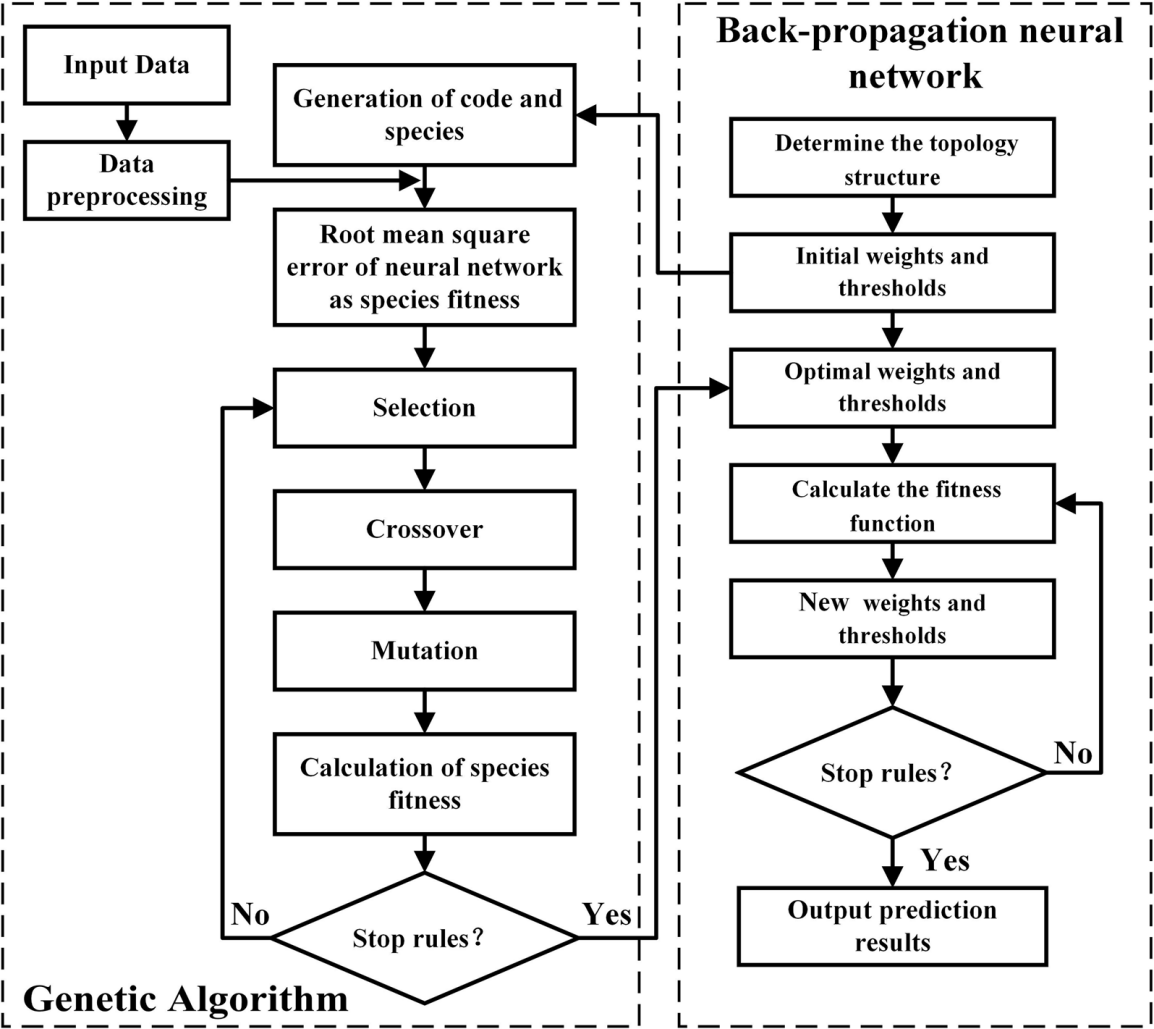
The flow chart of GA-BP

The back propagation (BP) neural network, first proposed by Rumelhart and McClelland [15], is a widely used algorithm for training feedforward neural networks for solving complicated and nonlinear problems. As shown in Fig. 1, a typical BP neural network consists of an input layer, a hidden layer, and an output layer, which was detailed described elsewhere [16]. It is known that the initial weights and thresholds most important parameters in BP neural network training, which are usually chosen randomly. It may lead to the BP neural network training falling to a local minimum and prolonging the train time. Genetic algorithm (GA) is widely used to generate high-quality solutions to optimization and search problems, which can be used to optimize the initial weights and thresholds of the BP neural network [17]. Hence, the combined GA-BP neural network model is used for predicting the size of the produced micro/nano-structures. Figure 2 shows the flow chart of the GA-BP neural network model and the detailed process is described by other literatures [18–20]. Before the input data and test data should be normalized before training.

### The release of Ag^+^

The amount of Ag^+^ released from the coated samples immersed in the phosphate-buffered saline (PBS) is monitored. After the samples are immersed in 6 ml of PBS at 37 °C for 1 day, the samples are taken out and immersed in 6 ml of fresh PBS again. This process is repeated 10 times to determine the Ag^+^ release time profile. The inductively-coupled plasma atomic emission spectrometry (ICP-MS, Thermo Fisher Scientific, USA) is employed to analyze the amount of released Ag^+^ in PBS solution.

### Bacterial culture

*E. coli* (ATCC25922) and *S. aureus* (ATCC25923) are employed to evaluate the antibacterial activity of the as-prepared samples. These bacteria are obtained from the Fourth Medical Center of PLA General Hospital. Bacterial culture is described in our previous study. The bacterial inoculation concentration was 10^5^ CFU/mL.

### SEM observation

The samples are put into a sterile 24-well plate, and 1 mL of bacteria suspensions is added to each well, so that the samples are completely immersed, and maintained at 37 °C under anaerobic conditions for 24 h. The samples with biofilm formation are lightly rinsed three times with 1 mL of sterile 0.89% sodium chloride solution, fixed with 2.5% glutaraldehyde at room temperature for 2 h, washed with ultrapure water, and then dehydrated in a series of ethanol solutions (30%, 50%, 70%, 80%, 90%, 95%, and 100%) for 10 min each. Finally, the samples are dried at room temperature for 24 h at least and sputtering-coated with gold. The biofilms are observed on a scanning electron microscope (SEM, SU8010, Hitachi, Japan). Three samples are used in this experiment (n = 3).

### Bacteria live/dead staining

*E. coli* and *S. aureus* are separately seeded on each sample and incubated for 6 h at 37 °C. After washing thrice with PBS, the bacteria on the samples are stained by a LIVE/DEAD BacLight Viability Kit (L-7012 LIVE/DEAD BacLight Bacterial Viability Kit, Thermo Fisher Scientific, USA) according to the instruction and observed by an epifluorescence microscope (TE2000-S2, Nikon, Japan).

### Anti-bacterial rate

Bacteria at 10^5^ CFU mL-1 have been cultured onto different samples for 6 h. After washing three times with PBS solution to remove the attached bacteria, the adherent bacteria on the samples were transferred to 1 mL PBS solution by ultrasonic vibration for 5 min. Then the 1 mL PBS solution is serially diluted by10^5^ times. After that 100 μL of the diluents were plated onto soy agar plates for overnight incubation at 37 °C. Finally, the bacterial colonies were counted according to the National Standard of China GB/T 4789.2 protocol.

The anti-bacterial rate is calculated according to the following equation:

1 $${\text{AR}} = \left( {{\text{A}} - {\text{B}}} \right)/{\text{A}} \times 100\%$$

where AR is the antibacterial rate, A is the mean number of viable bacteria on the original surface, and B is the mean number of viable bacteria on the laser-treated samples.

### Morphology of bacteria

After incubation for 6 h, 0.5 mL of glutaraldehyde solution (2.5 vol %) is used to immobilize the bacteria for 4 h. Then, the bacteria are dehydrated by using water and ethanol mixtures with volume fractions (ethanol vol %) 30, 50, 75, 90, 95, and 100%, respectively. After dehydration, the samples are dried. The morphologies of bacteria on samples are observed with the SEM.

### Cell culture

A mouse embryo osteoblast precursor (MC3T3-E1, Chinese Academy of Medical Sciences & Peking Union Medical College, Beijing, China) cell line is used to investigate the in vitro biocompatibility of the samples. MC3T3-E1 are cultured in alpha minimum essential medium (α-MEM) medium containing 10% fetal bovine serum (FBS). The cells are cultured in an incubator with air humidity of 95%, CO_2_ concentration of 5%, and temperature of 37 ℃.

### Hemolysis rate

Hemolysis rate experiments are conducted in accordance with national standards GB/T16886 and GB/T 16175-2008. The as-received sample and prepared hierarchical micro/nano-structures are ground into powder. Then the powder is mixed with normal saline according to the standard 5 g/10 ml. The mixed solution is immersed in 37 ℃ water for 30 min. The normal saline (Aladdin, Shanghai) and distilled water are chosen as negative and positive control, respectively. 8 ml New Zealand rabbit blood (Sigma-Aldrich, America) is diluted with normal saline 10 ml followed by 5% EDTA (Aladdin, China). Take 10 ml leaching liquor from the sample group, negative control group, and positive control group, respectively, then 0.2 ml diluted rabbit blood is added to each tube. All tubes are immersed in 37 ℃ water for 30 min, then the tubes are centrifuged by 1500 r/min for 5 min. The supernatant is taken into the cuvette and a UV spectrophotometer (UV2550, SHIMADU, Japan) is used to detect the absorbance at the wavelength of 540 nm. Hemolysis rate is estimated as the following calculation: [21]

2 $${\text{Hemolytic percentage}}\;\% = \frac{{D_{t} - D_{nc} }}{{D_{pc} - D_{nc} }} \times 100\%$$

where the D_t_ and D_nc_ are the absorption of the test sample and negative control group, respectively. The D_pc_ is the absorption of the positive control group.

### SEM observation

The detailed cell viability and cell morphology test are described in our previous study [22, 23].

### Statistical analysis

For all bacteria and cell experiments, three independent samples are tested for each experimental group. For each sample, three technical replicates are performed. All data are presented as mean ± standard deviation values, and the standard deviations are plotted as error bars in all figures.

## Results and discussion

### Micro/nano-structure evolution

**Fig.3:**
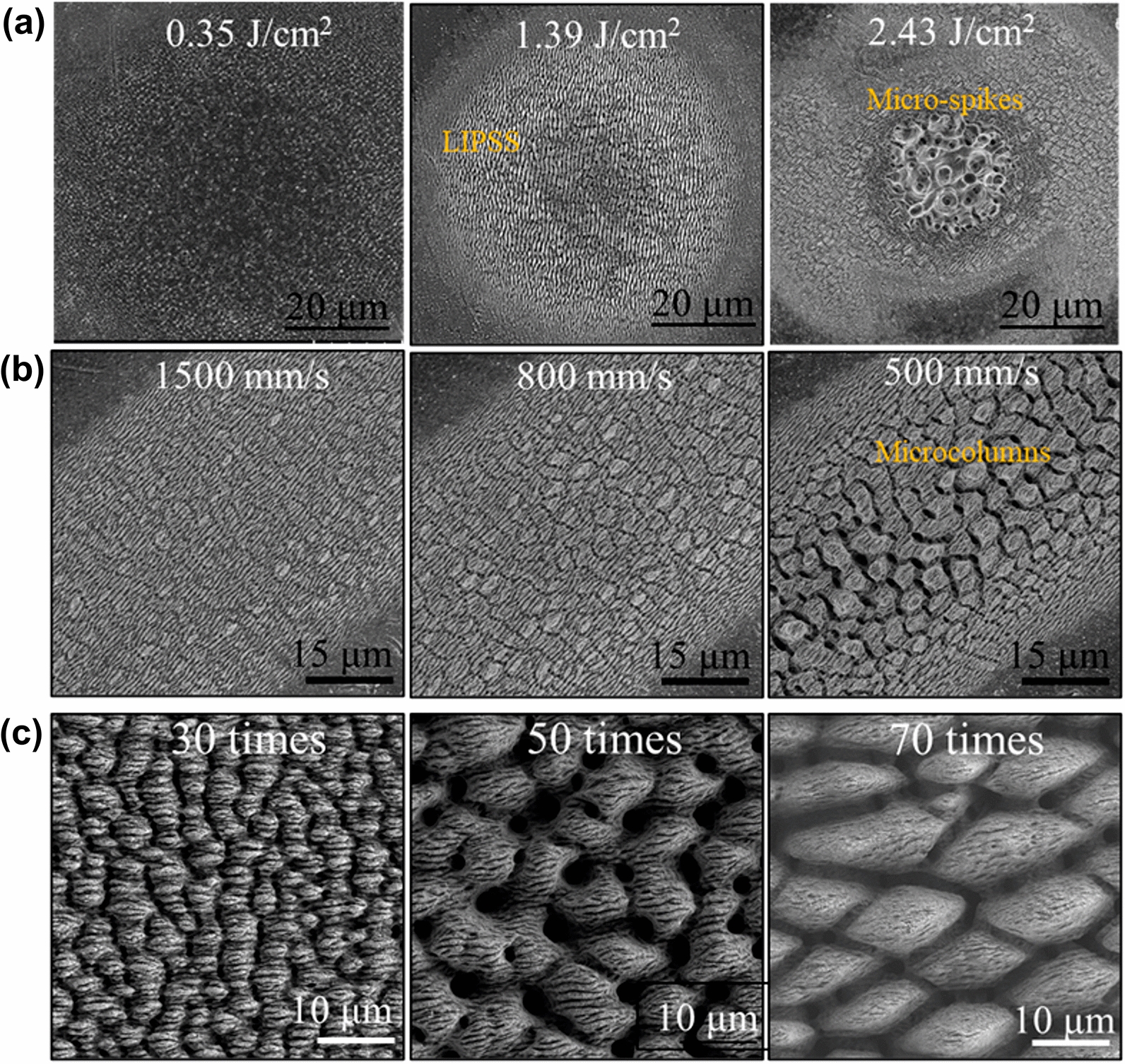
Micro/nano-structure evolution. **a** Femtosecond laser single irradiated spots on Ti6Al4V with different radiation fluence: 0.35 J/cm^2^, 1.39 J/cm^2^, and 2.43 J/cm^2^. **b** The morphological evolution from LIPSS to micro-cone on Ti6Al4V by femtosecond laser single line irradiation (0.7 J/cm^2^, 300 kHz, 10 times) with the scan speed decreasing. **b** Evolution of the size of micro-cone by femtosecond laser parallel irradiation (0.7 J/cm^2^, 300 kHz, 1000 mm/s) with different scan times (30, 50, and 70, respectively)

Figure 3a shows the morphological evolution from nanoparticles (NPs) to laser-induced periodic surface structures (LIPSS) on Ti6Al4V by femtosecond laser irradiation. At relatively low laser fluence of about 0.35 J/cm^2^, the Ti6Al4V surface is covered by a random distribution of NPs. When laser fluence increased up to 1.39 J/cm^2^, LIPSS is formed with a decreasing number of NPs. At relatively high laser fluence of 2.43 J/cm^2^, LIPSS in the central area of the irradiated spot evolve into micro-spikes. During the laser-material interaction, the photon energy is first absorbed by the conduction electron, and then transfer to the lattice through electron-lattice coupling [24]. The surface experiences a rapid transformation into solid, liquid, gas, and plasma. It has been demonstrated that the formation of NPs is attributed to the nucleation of plasma generated in the phase explosion and coagulation of gas [25]. With the laser fluence increasing, the dispersed NPs start to agglomerate on lines leading to the formation of LIPSS structures. When the laser energy is too high, the LIPSS structure will be destroyed (see micro-spikes in the central area of the irradiated spot).

Figure 3b shows that LIPSS gradually evolved into micro-cone on Ti6Al4V by femtosecond laser single line irradiation (0.7 J/cm^2^, 10 times) with the scan speed decreasing. Figure 3c shows the evolution of the size of microcolumns by femtosecond laser parallel irradiation (0.7 J/cm^2^, 1000 mm/s) with different scan times (30, 50, and 70, respectively). The micro-cone grows in height and width and tends to merge as growth proceeds as the laser scan times increase. This may be caused by the splashing and flow of molten material between the micro-cone [26].

### Parameter optimization by neural network

As illustrated in Sect. 3.1, there are mainly two different types of structures with the femtosecond laser, namely LIPSS and micro-cones (MCs). Moreover, MCs contain two structures of different sizes, namely MCs1 and MCs2. As shown in Fig. 3, the LIPSS is a ripple structure with particular width and period, while the MCs are triple-scale hierarchical structures, which consist of submicron-scale ripples, micron-scale cones, and nano-scale particles. The characteristics of structures are influenced by the laser processing parameters. To describe the relationship between the characteristics of structures and the laser processing parameters, the network structure of 4 × 10 × 4 GA-BP is employed to establish the non-linear neural network model. The input layer has four nodes corresponding to the four main laser processing parameters (power, repetition rate, scan times, and scan speed), and the period and width of LIPSS (PL, WL), diameter and period of MCs (DM, PM) are considered as the output layer. In this work, the average laser power and repetition rate are adjusted in the range of 1 W–3 W and 100 kHz–400 kHz, respectively, and the laser scan times and scan speed are chosen to be between 10 to 70 and 500 mm/s to 2000 mm/s, respectively. These input values are summarised in Table 1. Hence, as listed in Additional file 1: Table. S1, 256 experimental data are obtained from the experiments. Among the 256 experimental results 200 experimental data are randomly chosen as training samples and the rest of the 56 sample data are chosen as testing samples. It should be noticed that when the diameter and period of MCs are 0, the surface structure is LIPSS.

**Table 1:** The input of laser parameters

Laser parameters	Chosen value			
Power (W)	1	2	3	4
Repetition rate (kHz)	100	200	300	400
Scan times	10	30	50	70
Scan speed (mm/s)	500	800	1500	2000

**Fig.4:**
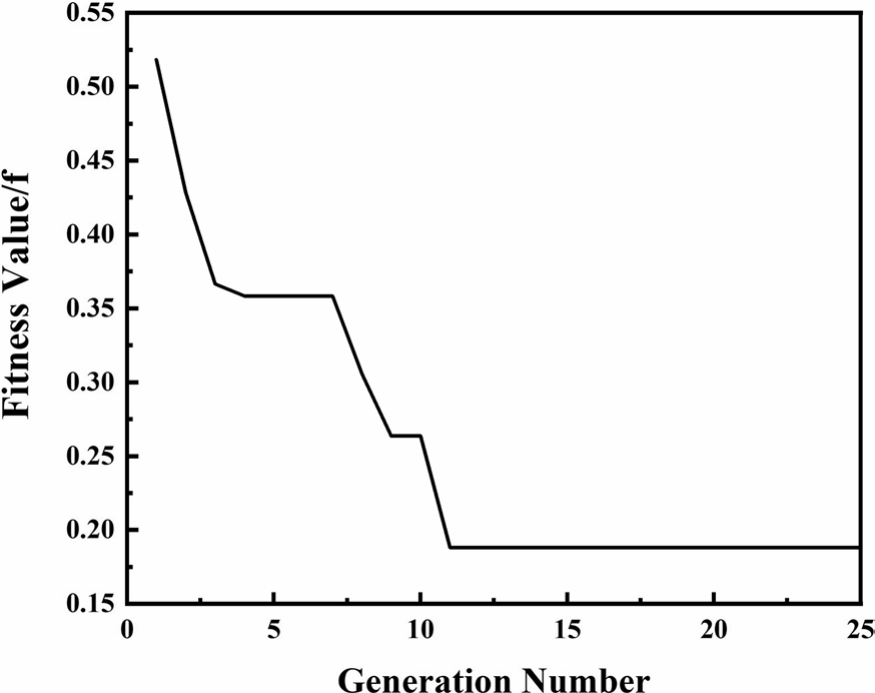
Evolution of fitness function in GA-BP neural network

**Fig.5:**
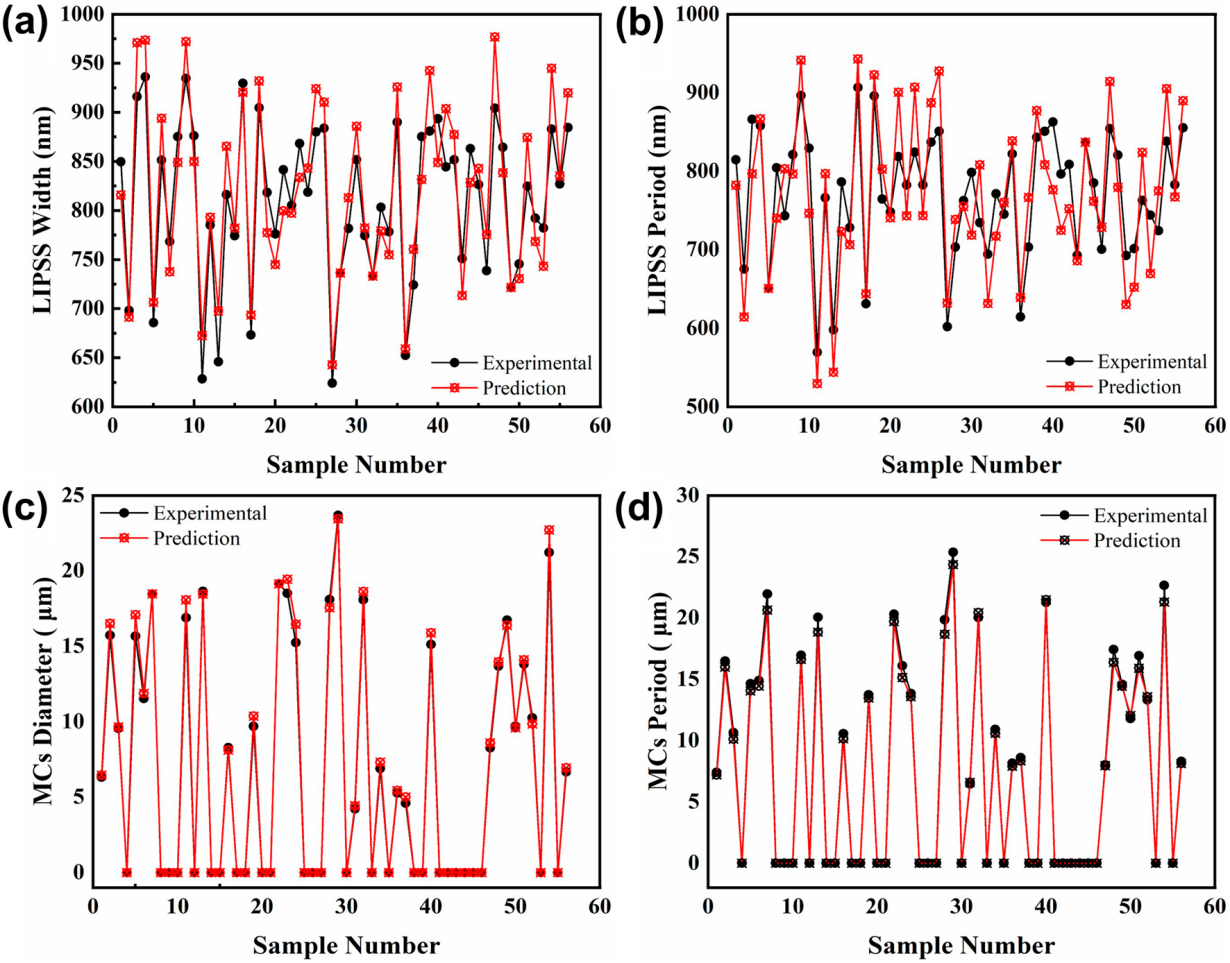
Comparisons between experimental values and prediction values and for training samples. **a**, **b** period and width of LIPSS; **c**, **d** diameter and period of MCs

**Fig.6:**
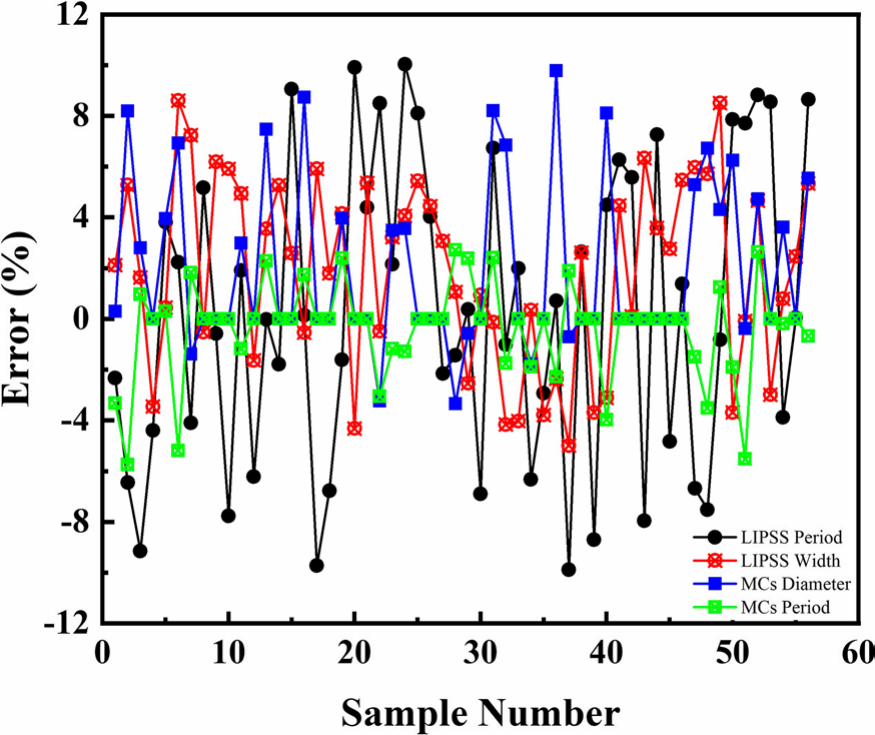
Training error of GA-BP neural network model

Figure 4 presents the evolution of the fitness function in the GA-BP neural network. It can be seen that the fitness function value reaches 0.185 after the population is successfully inherited to the 11th generation. The experimental and prediction values for training samples are shown in Fig. 5. Figure 6 shows the training error of the GA-BP neural network and the error of the experimental values and prediction values is below 10%. This indicates that the GA-BP neural network model developed in this work has good predictive capability and can be used for controlling the size of surface structures.

### Surface characterization

**Fig.7:**
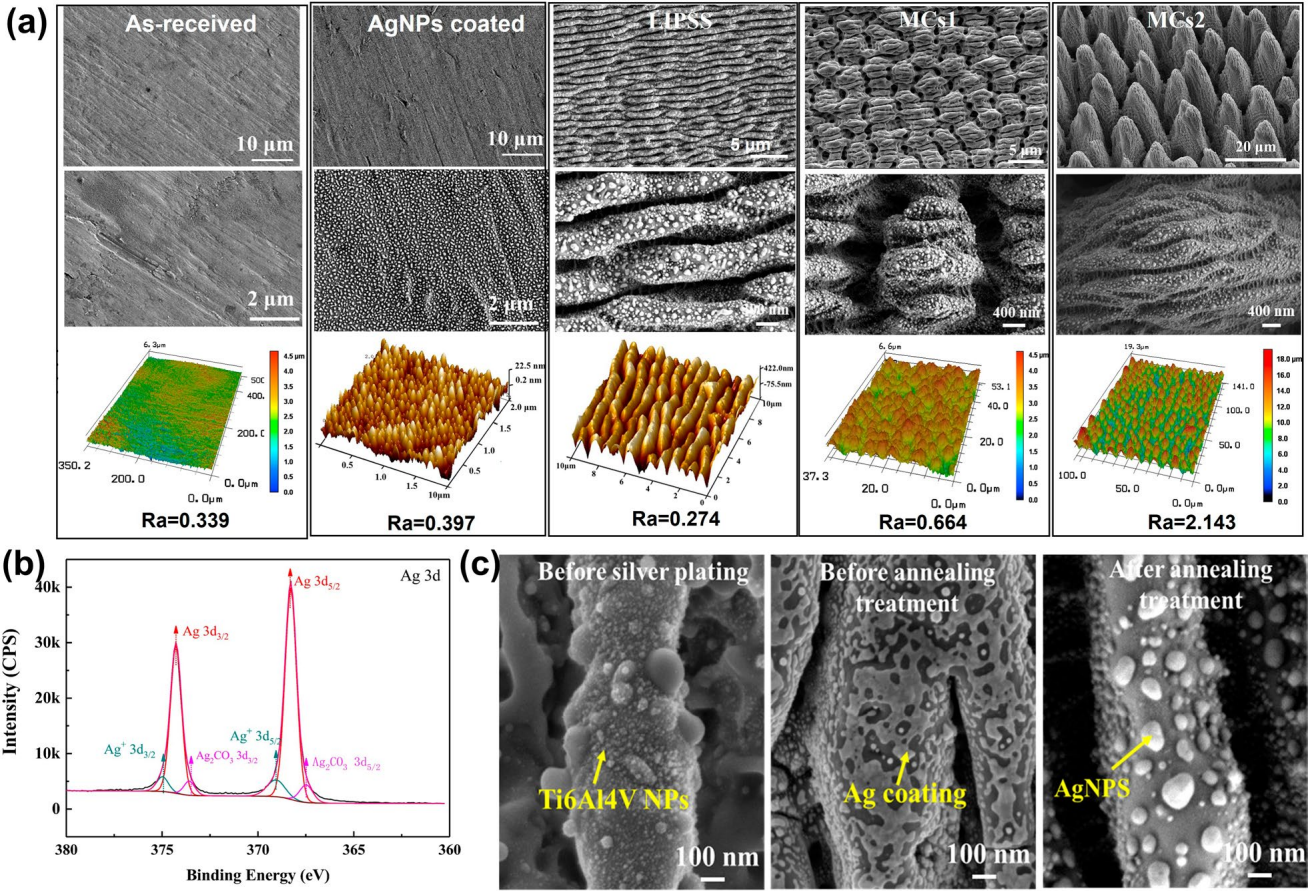
Surface characterization. **a** SEM and AFM images of as-received, AgNPs coated, LIPSS, MCs1 and MCs2 samples. **b** XPS spectra of AgNPs. (**c**) SEM images of the evolution of the Ag coating

To find the hierarchical micro/nano structures with the best synergistic effect with AgNPs, we fabricate two different types of structures with the fs laser based on the GA-BP neural network. As-received and AgNPs coated samples are used as the control. As shown in Fig. 7a, The LIPSS is a two-scale hierarchical structure consisting of submicron-scale ripple and nano-scale particles, while the MCs1 and MCs2 are triple-scale hierarchical structures consisting of submicron-scale ripples, micron-scale cones, and nano-scale particles. The surface roughness (Ra) of the five surfaces is 0.339 μm, 0.397 μm, 0.274 μm, 0.664 μm and 2.143 μm, respectively. The average period and depth of the submicron ripples are 850 nm and 200 nm, respectively. The average period and depth of MCs1 and MCs2 are 2.5 μm and 2.5 μm, 10 μm and 6 μm, respectively. The NPs with the diameter ranging from 5 to 80 nm are randomly distributed on the surface of the structures.

XPS shows that the main component of NPs is Ag, which exists in the form of Ag_2_CO_3_, as shown in Fig. 7b. The evolution of the Ag coating is illustrated in Fig. 7c. It can be seen that the surface is covered with Ti6Al4V NPs before Ag deposition, then a discontinuous Ag film is formed on the surface after Ag deposition. The Ag film changed into AgNPs after the annealing treatment, which is driven by the surface energy differences [27]. The core of AgNPs may be the Ti6Al4V NPs. Therefore, the AgNPs prepared in this work can be firmly fixed on the Ti6Al4V substrate, which not only prevents the aggregation of AgNPs but also reduces the deposition of AgNPs in the human body.

### Surface wettability

**Fig.8:**
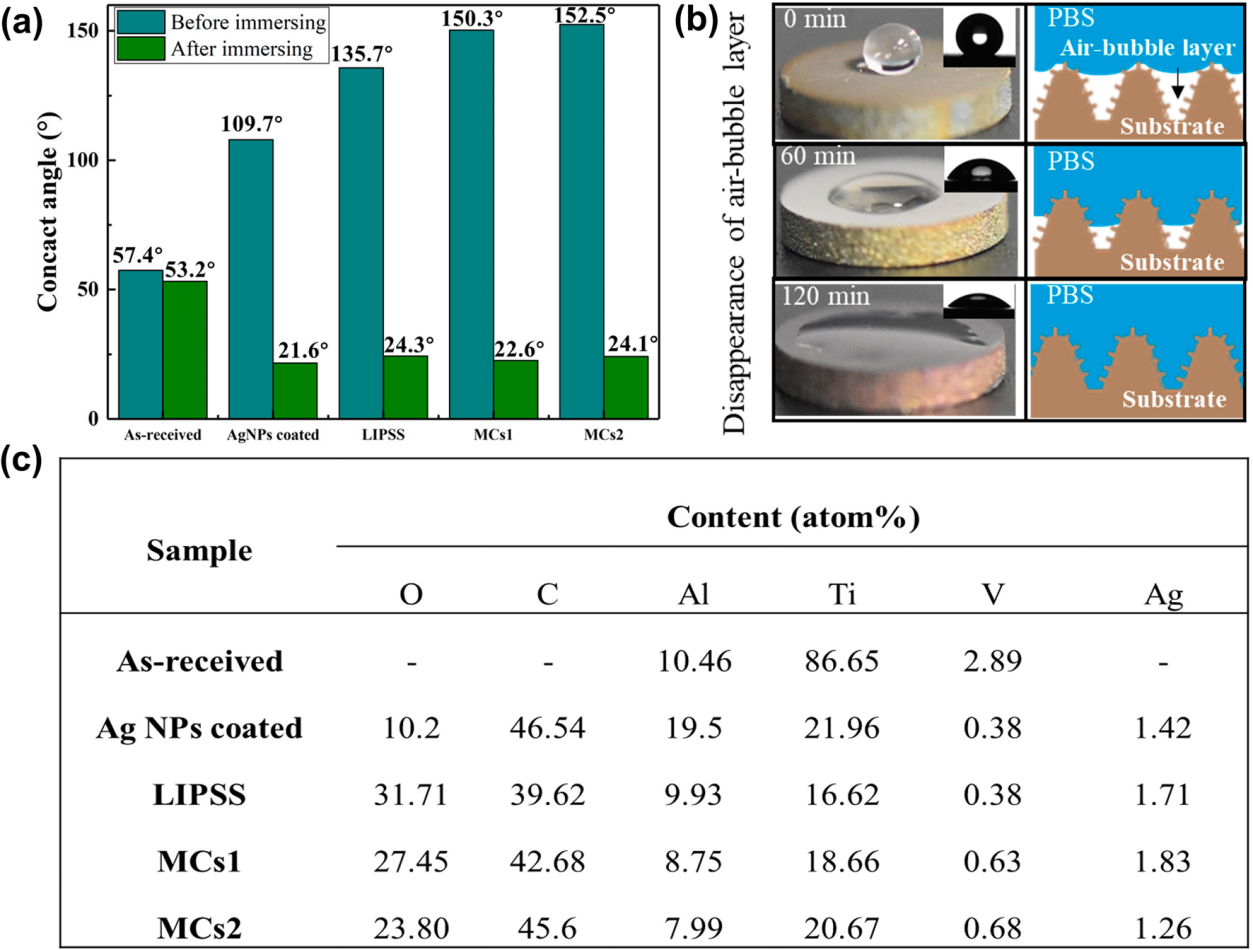
Surface wettability. **a** The contact angle before and after immersing in PBS solution. **b** During the immersing process, the wetting performance changes with the disappearance of the air-bubble layer. **c** Surface chemical composition

The surface wettability affects bio-functions such as bacteria/cell adhesion and spreading [28, 29]. As shown in Fig. 8a, the contact angle of the five samples is 57.4°, 107.9°, 135.7°, 150.3°, and 152.5°, respectively. The synergy of hierarchical micro/nano-structures and AgNPs drastically increase the contact angle. The change in surface wettability is mainly related to the topography and the chemical composition. On the one hand, the submicron-ripple and AgNPs of the LIPSS surface increase the interfacial area between solid and liquid, resulting in the LIPSS surface being in the Wenzel state [30]. For the MCs surface, the topography of double roughness on top of micro cones is very beneficial to trap air pockets beneath the water droplet, resulting in the MCs samples in the Cassie–Baxter state [31]. On the other hand, the C content on the structured surface significantly increases because of the absorption of non-polar organic compounds, as shown in Fig. 8c, which can also improve the hydrophobicity [32].

Considering that the implant is used in a liquid environment, we further investigate the wettability of samples immersed in PBS. After immersing in PBS solution for 2 h, the contact angle decreases to 53.2°, 21.6°, 24.3°, 22.6°, and 24.1°, respectively, as shown in Fig. 8a. This phenomenon indicates that the hydrophobicity is unstable under the liquid environment for the structured surface. The reason may be that the liquid can gradually permeate into the asperities and the air-bubble layer is expelled during the PBS immersing, resulting in the transformation of hydrophobicity to hydrophilicity, as shown in Fig. 8b.

### Ag^+^ release

**Fig.9:**
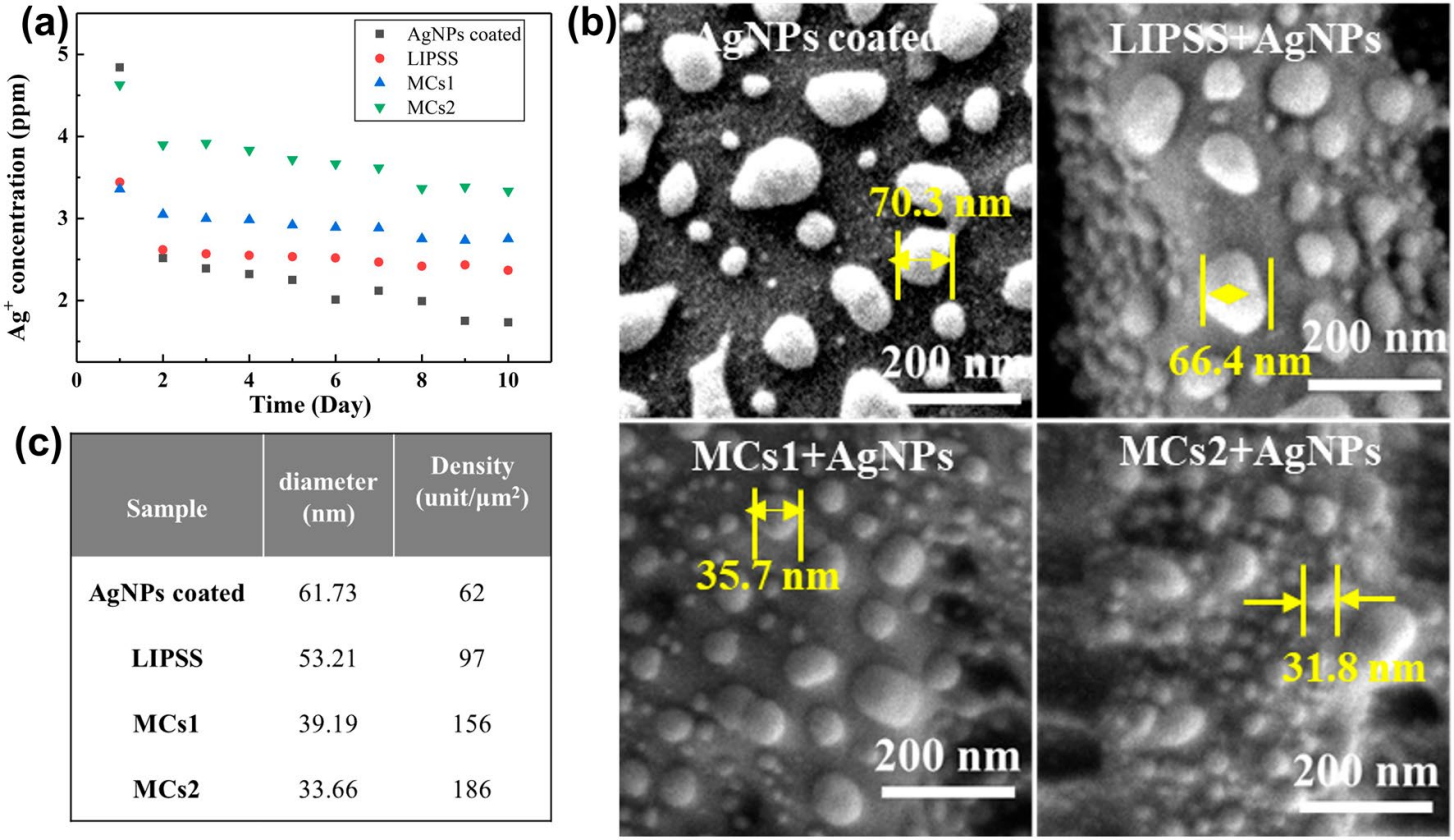
Ag^+^ release. **a** Ag^+^ release profiles from AgNPs into PBS solution. The evolution of the silver coating. **b** SEM images of AgNPs on the four samples surfaces. **c** Dimensional feature measurements of AgNPs

Figure 9a shows the Ag^+^ release time profiles from AgNPs into PBS solution. The results show that amount of released Ag^+^ from AgNPs coated sample is much larger on the first day, which will lead to short-term bactericidal performance. For LIPSS, MCs1, and MCs2 samples, the amounts of released Ag^+^ remained relatively flat. This may be because of the establishment of the superhydrophobic surface of the structured samples, which can help to prevent the initial burst release of Ag^+^. In addition, the average release rates of Ag^+^ from the four samples are different, which is in the following order: AgNPs coated sample < LIPSS sample < MCs1 sample < MCs2 sample. This difference is induced by the different sizes and densities of AgNPs on the four surfaces, as shown in Fig. 9b and c. It is worth mentioning that the Ag^+^ release levels in all samples are all lower than the maximum toxic concentration for human cells (10 ppm) and higher than the minimum concentration required for antimicrobial efficacy (0.1 ppm) [33]. Hence the Ag^+^ release rate meets clinical requirements.

### Hemolysis rate

**Fig.10:**
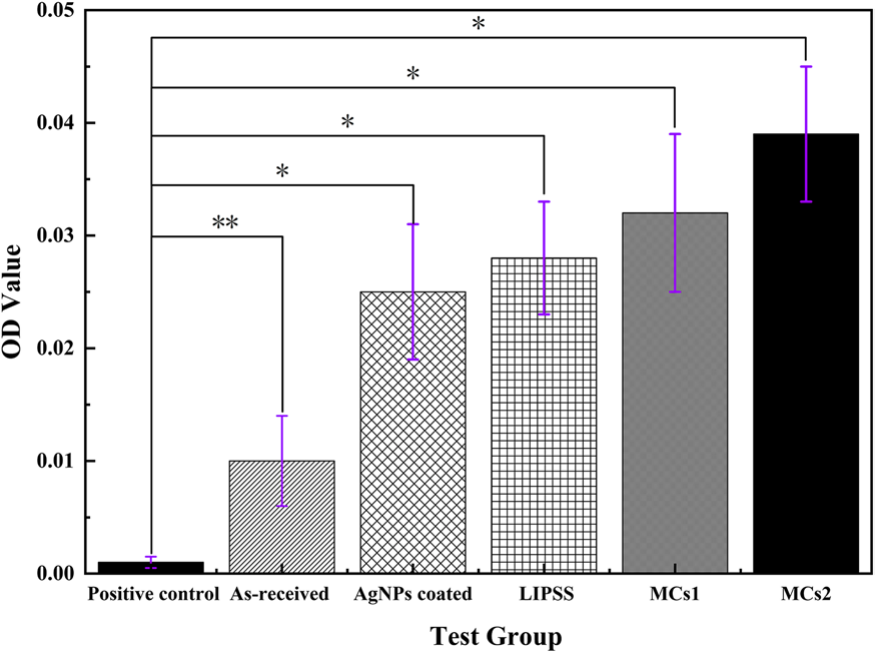
OD value at the wavelength of 540 nm for different samples. *P < 0.01, **P < 0.05

The hemolytic test is used to determine the red blood cell dissolution and the hemoglobin dissociation degree, which is an important property of the implant materials. The higher the hematolysis rate, the worse the blood compatibility of materials. The OD value at the wavelength of 540 nm for different samples is shown in Fig. 10 and the corresponding hemolysis rate is listed in Table 2. It can be seen that the hemolysis rates of AgNPs coated samples including LIPSS, MCS1, MCS2 are 3.01%, 3.38%, 3.88%, and 4.76%, respectively. Although the hematolysis rate becomes higher after Ag deposition compared to that of the as-received sample. It is noted that the hemolysis rate of all Ag-coated samples is still within the permissible range of biomaterials as less than 5% [34]. Therefore, the present Ag-coated samples have good blood compatibility and reach the requirements of security for the biological materials.

**Table 2:** Hemolysis rate of different sample

Sample	Hemolysis rate (%)
As-received	1.13
AgNPs coated	3.01
LIPSS	3.38
MCs1	3.88
MCs2	4.76

### The hierarchical micro/nano-structures promote osteoblasts growth

**Fig.11:**
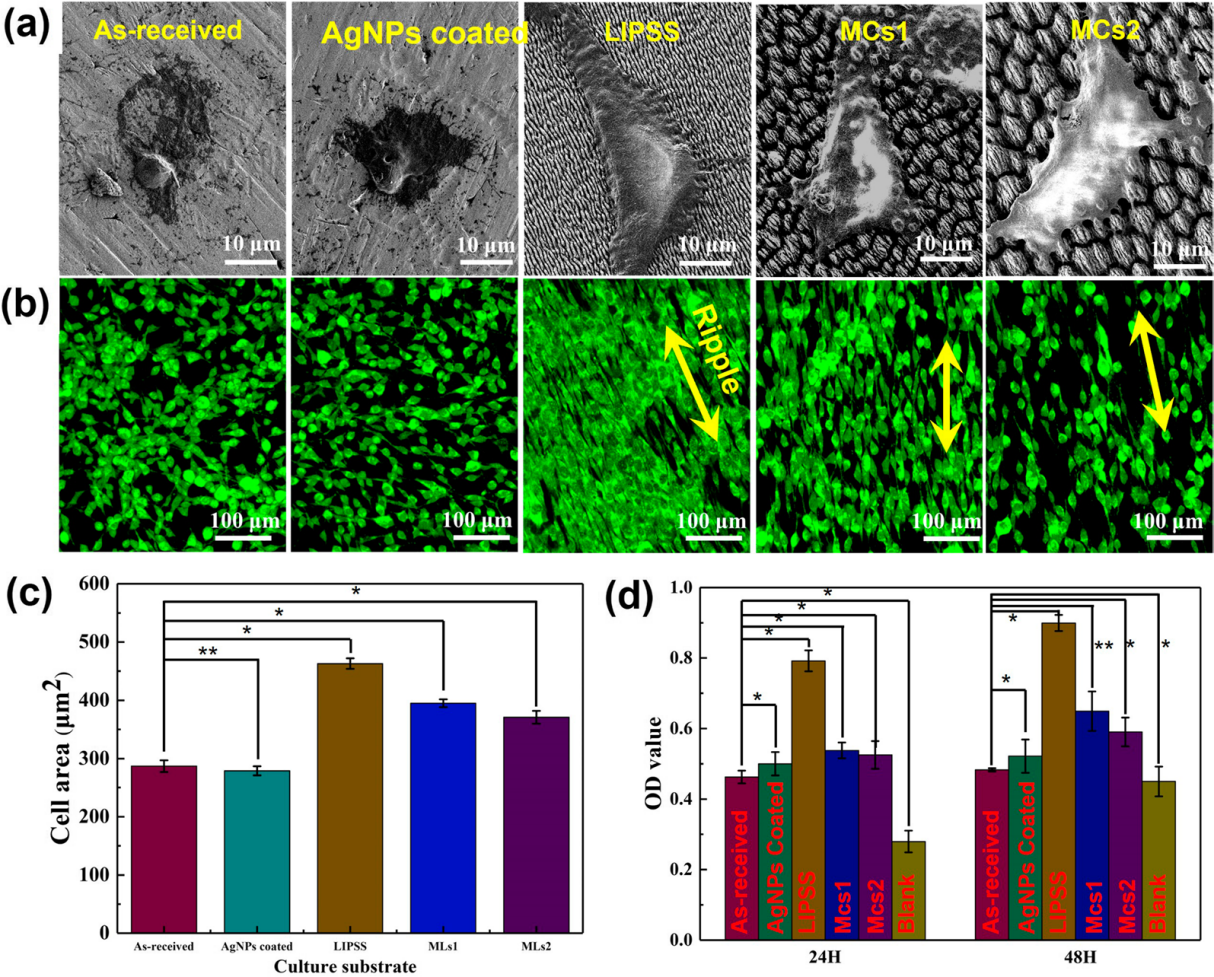
Effects of surface morphology on cell. **a** SEM images of cell cultured for 24 h. **b** Fluorescence images of cell cultured for 24 h. **c** The average cell area on the five samples surfaces at 24 h after cell seeding. **d** The OD value of cell cultured for 24 h and 48 h. *P < 0.01, **P < 0.05

Figure 11a shows the attached cells are observed by SEM after being cultured for 24 h. It can be seen that the cells on the as-received and AgNPs coated sample is still spherical, while on the three structured surfaces, cells are more voluminous and spread out. Figure 11c presents the statistical value of the cell spreading area, which indicates that the structured surface is beneficial to the osteoblasts spreading. In addition, more filopodia can be found on the leading edge of the stretched cells cultured on the three structured surfaces. The cell distribution is further observed by fluorescence images in Fig. 11b. The shape of the cells tends to elongate along the direction of submicron-ripple on the three structured surfaces. The cell proliferation evaluation is evaluated using the CCK-8 array after the cells are cultured for 24 h and 48 h on the different samples, as shown in Fig. 11d. On the as-received and AgNPs coated sample, there is no significant difference in the cell proliferation, indicating that the AgNPs has no obvious negative effect on the cells. The cells grown on the three structured surfaces are higher than that of on the as-received sample. Especially on the LIPSS surface, the OD value is 1.8 times higher than that of on the as-received sample. The results indicate that the hierarchical micro/nano-structures can improve cell adhesion, spreading performance, and proliferation capacity, which can attribute to the contact guidance of the submicron ripple [35]. As a mechanical constraint, submicron ripple can change cell morphology, cytoskeleton, and control cells to secrete richer extracellular matrix to promote cell proliferation [36].

After further comparing the cell on the three structured surfaces, the cell proliferation capacity is in the following order: LIPSS > MCs1 > MCs2. Cell activity decreases with the increase of the complexity and size of the structures. This may be because the high vertical depth of the micro-cone on the MCs + AgNPs sample forms an energy barrier. The energy barrier increase with the depth increases from 2.5 μm to 6 μm. The high energy barrier hinders the focal adhesion formation and the actin polymerization, which restricts the filopodia formation and cell spread [37]. Therefore, the LIPSS and MCs1 structure can better improve the cytocompatibility than the MCs2 structure.

### The synergy of hierarchical micro/nano-structures and AgNPs improves the antibacterial ability

**Fig.12:**
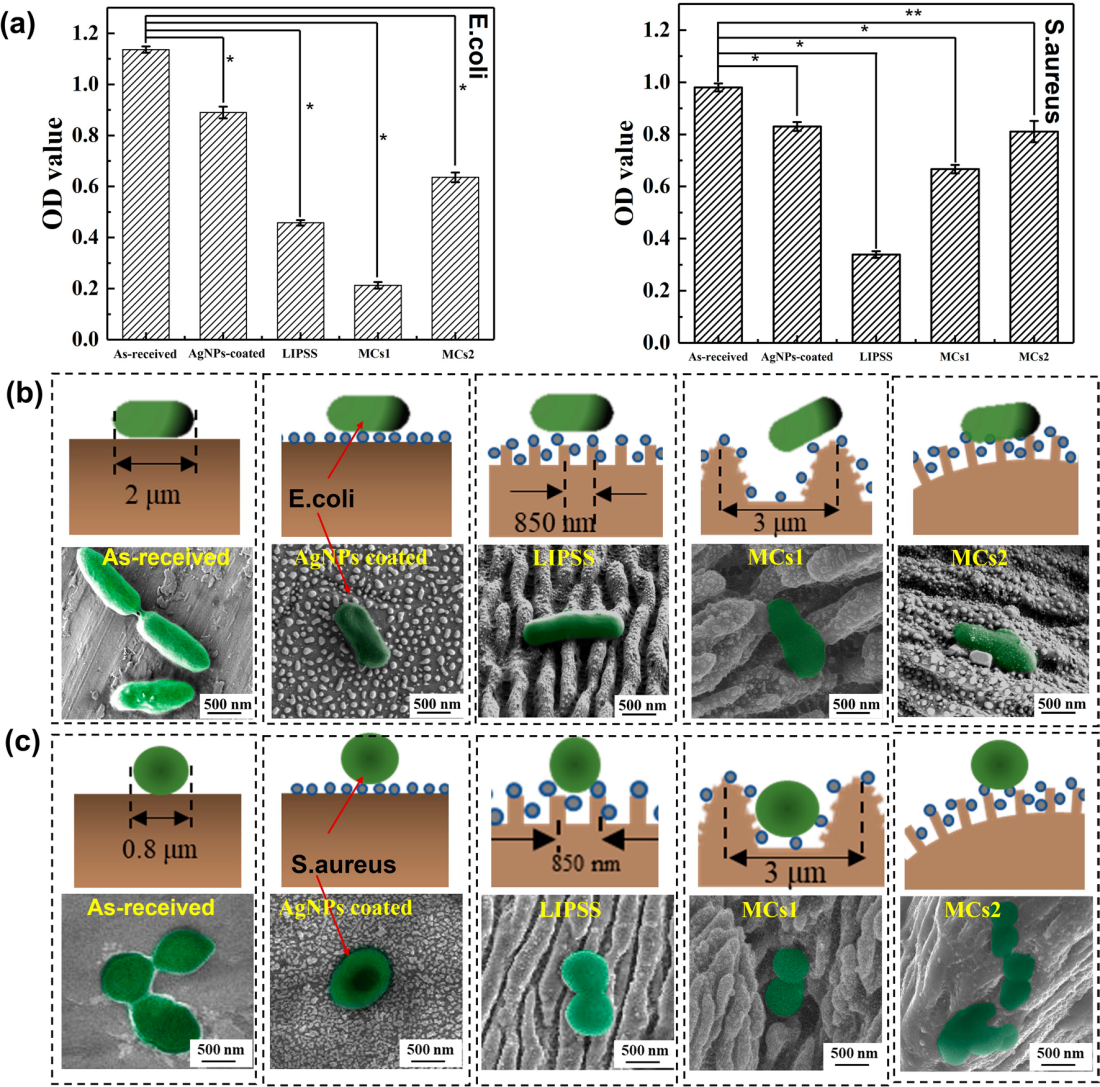
Influence of the size of hierarchical micro/nano-structures on initial bacterial adhesion. **a** OD value of initial bacterial adhesion. **b** The surface contact state between *E. coli* and each sample. **c** The surface contact state between *S. aureus* and each sample. *P < 0.01, **P < 0.05

To explore the effect of hierarchical micro/nano-structures on bacteria adhesion without the influence of surface wettability, we first immerse all the samples in PBS solution until surfaces have similar contact angles. Then the bacteria are incubated for 30 min, and the planktonic bacteria on the surfaces are washed off with PBS. Average absorbance is used to detect the amount of remaining viable bacteria, as shown in Fig. 12a. For *E. coli*, the poorest adhesion is on the MCs1 surface, while for *S. aureus*, it is the LIPSS surface. The results indicate that bacterial adhesion not only depends on the surface characteristics but also on the bacteria type. As shown in Fig. 12b and c, *E. coli* with a dimension of about 2.0 μm long and 0.5 μm in diameter are rod-shaped cells, while the *S. aureus* are spherical cells with diameters of about 1 µm. On the MCs1 surface, both the period and height of the micro- cone are about 2.5 μm, which is similar to the size of *E. coli*. So, due to the high rigidity of *E. coli*, it is difficult for the bacteria to adjust their cell shape to fit the complex 3D topography. For *S. aureus*, the diameter of the cell (about 800 nm) is almost similar to the peak-to-peak distance of submicron ripples (about 850 nm), which cannot provide sufficient contact points for *S. aureus* to adhere. On the MCs2 surface, the dimension of the micro-cone is much larger than both *E. coli* and *S. aureus*, so a single micro-cone in the MCs2 surface can offer multiple adhesion faces for bacteria. Therefore, the MCs2 structure is not conducive to inhibiting bacterial adhesion. In summary, when the size of hierarchical micro/nano-structures is similar to bacteria, it can effectively decrease the contact area with bacteria and reduce bacterial adhesion, while the hierarchical micro/nano-structures, which are much larger than the bacteria, can increase the specific surface area and improve bacterial adhesion.

**Fig.13:**
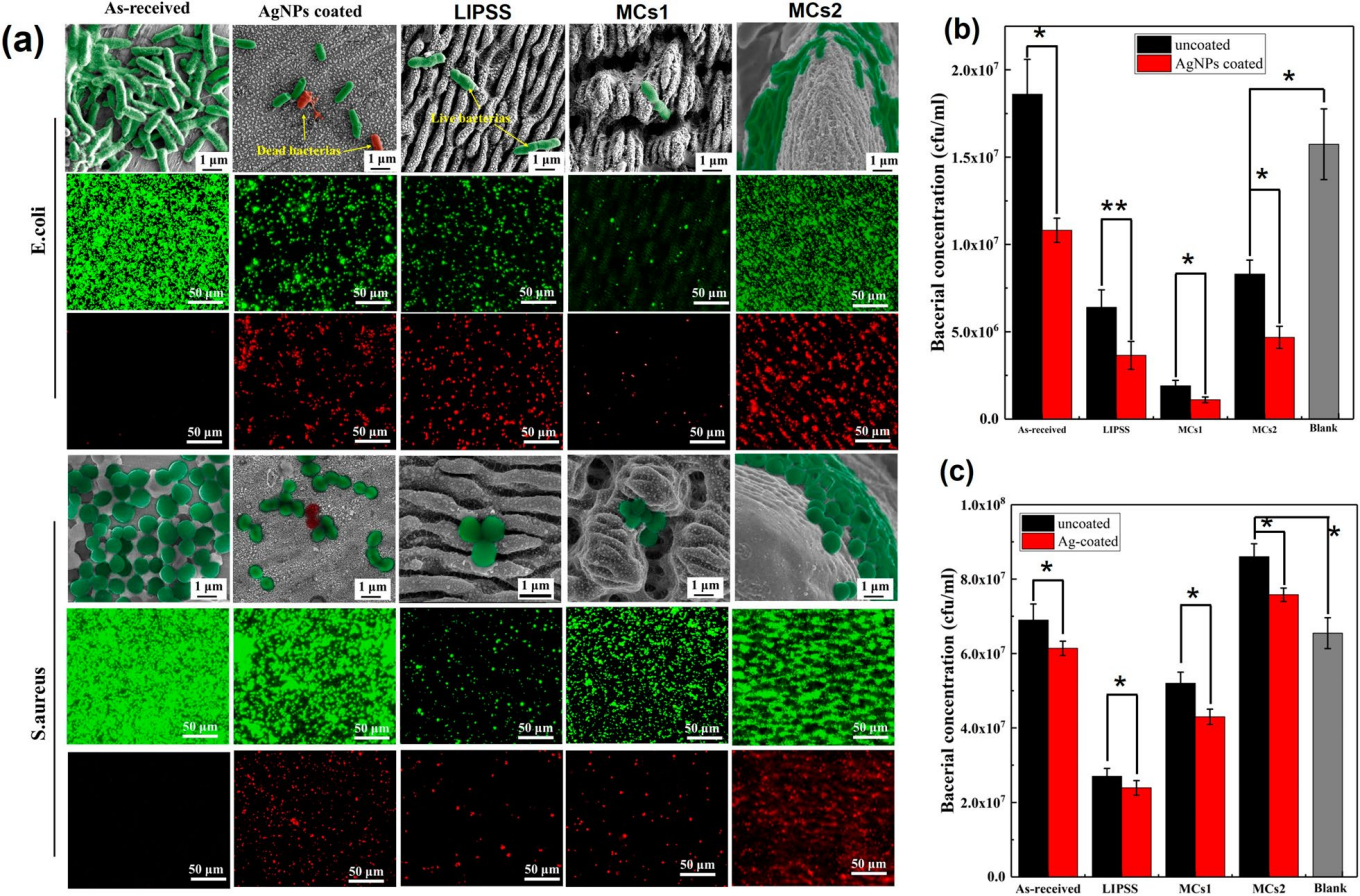
Antibacterial property. **a** SEM and fluorescent images of the live/dead staining of *E. coli* and *S. aureus* after culturing for 6 h. **b** Bacteria concentration of *E. coli*. **c** Bacteria concentration of *S. aureus*. *P < 0.01, **P < 0.05

In order to further investigate the antibacterial effect of the prepared surface, the duration of bacterial culture is extended to 6 h. The bacteria morphology is shown in Fig. 12a. Compared with the as-received surface, the morphologies of both *E. coli* and *S. aureus* on the four AgNPs coated samples including as-received, LIPSS, MCs1 and MCs2 samples are smaller and irregular. This is probably due to the low bacterial activity caused by AgNPs. This is further confirmed by the fluorescent images of live/dead staining, as shown in Fig. 13a. Almost no dead bacteria (dyed red) are found on the as-received sample surfaces. Plenty of dead *E. coli* and *S. aureus* (dyed red) can be found on the four AgNPs coated samples, indicating that the prepared AgNPs have good antibacterial activity. The covered AgNPs can continuously release the Ag^+^, which can improve the permeability of the cell plasma membrane, leading to the destruction of the cell membrane of bacteria [38]. The AgNPs with a diameter of less than 10 nm can penetrate the bacterial cell wall and cause further damage[39].

The antibacterial rates of various surfaces are characterized by plate count. According to the bacterial concentration of *E. coli* and *S. aureus* showed in Fig. 13b and c, the corresponding antibacterial rates of AgNPs coated, LIPSS, MCs1 and MCs2 samples against *E. coli* can be estimated as 46.2%, 72.6%, 89.27% and 63.44%, respectively, while the antibacterial rates against *S. aureus* can be estimated as 11%, 65.4%, 37.7% and 9.9%, respectively. The difference in the antibacterial behavior of *E. coli* (89.27% with MCs1) and *S. aureus* (65.4% with LIPSS) may be caused by the different types of bacteria. On the one hand, the composition of structure is different to the cell wall, which makes the AgNPs easier to break through the Gram-negative bacteria [40]. On the other hand, for *E. coli*, it is the MCs1 surface, and for *S. aureus*, it is the LIPSS surface, that form a narrow and limited living space for bacteria, which can prevent the cells from physically (i.e., contact-dependent inhibition) and chemically (i.e., quorum sensing) interaction with one another [41]. The state of being imprisoned makes interference with bacterial behaviors, including adhering, elongating, and proliferating.

### Mechanism explanations

It is known that both the mechanism bio-mechanical and Ag-chemical can be used to realize biocidal activity. The mechanism of bio-mechanical biocidal is that micro/nano-structure with sharp edges induces the formation of pores by physical insertion into the bacterial membrane, which will cause osmotic imbalance and the death of the bacteria [42–44]. Meanwhile, the Ag-chemical biocidal mechanism is that when Ag^+^ reach the surface of the microbial membrane, it will enter the bacterial cells through a water-filled channel called porins in the outer membrane of the bacteria. After the Ag^+^ penetrates into the cells, it will attack cellular structures and biomolecules such as proteins, lipids, and DNA, thus damaging the internal structure of the bacteria, and leading to the death of bacteria [38, 45].

In fact, the micro/nano-structure produced by femtosecond laser in our work cannot kill the bacteria through the bio-mechanical biocidal, because the dimension of produced structures is sub-micron and micron, which is impossible to physically insert into the bacterial membrane. The bacteria ia killed through the Ag-chemical biocidal, while the fabricated micro/nano-structures can form a narrow living space for the bacteria, which can prevent cells from physically and chemically interacting with each other. Correspondingly, the bacterial behaviors including adhesion, elongation, and proliferation are inhibited finally.

The above results prove that the combination of surface structure and chemical surface functionalization is an effective way to manipulate the interaction between cells, bacteria, and implant surface. But few studies have reported using surface structure to promote cell growth and resist bacterial adhesion at the same time. In this work, we verify the effects of surface microstructure with different types and sizes on cell growth, and the results show that the directional surface structures can promote osteoblast attachment and guide cell growth compared to the smooth surface. Previously studies have shown that the cell cytoskeleton and the gene expression relating to cell adhesion, and can be changed by extracellular stimulus [46–48]. When cells perceive the physical or chemical properties of the implant surface, mechanical conduction signals will be generated inside the cells [49, 50]. Different conduction signals can regulate cell morphology by affecting cytoskeleton tissue, cell membrane protrusion, cell contractility, adhesive spots, and stress fiber stability, which are crucial to promote bone integration, including cell proliferation and mineralization [51–53].

**Fig.14:**
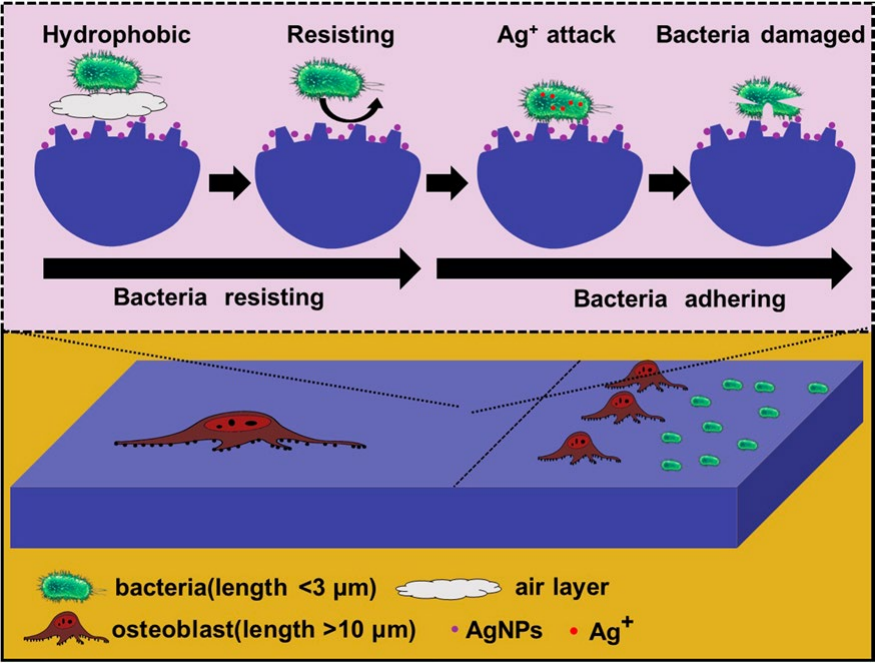
Schematic of controlling cell/bacteria on structured surface

Besides promoting bone integration, the antibacterial property of implants is another important issue in the prevention of implant infection. Previously results show that there are three stages of the adhesion of bacteria onto the material surface, which are transport, initial adhesion, and final attachment [54]. The second stage occurs in 1–3 h after the contact between the material surface and the bacterial suspension, which is mainly affected by the physical properties of the material surface, such as wettability and surface topologies [55]. From the wettability results, the structured samples are hydrophobic, which is difficult for bacteria to penetrate the air layer between the material and bacterial suspension and adhere to the material surface [56]. As shown in Fig. 14, hydrophobic property suppresses bacterial interaction with material surfaces at the early stage. However, it cannot rely on hydrophobicity to achieve long-term antibacterial properties. With the hydrophobic property deteriorating, bacterial suspension gradually contacts the material surface. At the same time, the surface topology began to resist subsequent bacterial adhesion. Meanwhile, Ag^+^ released from AgNPs will kill the bacteria both in suspension and attached on the material surface. The results of wettability and antibacterial rate show that hydrophobic property is not the deciding factor to inhibit bacterial adhesion. In this work, surface topologies are the key to the creation of the antibacterial surface. The size of hierarchical micro/nano-structures is similar to *E. coli* and *S. aureus*, which can effectively reduce the contact area and living space of bacteria to achieve antibacterial properties.

For clinical implants, it is critical to balance the cytocompatibility and antibacterial properties. Benefiting from size discrepancies between osteoblasts and bacteria (about 10 times the size of bacteria), the hierarchical micro/nano-structures used to imprison bacteria in this work cannot inhibit the cell behaviour. On the contrary, due to the mechanical stimulation, cell spreading area, geometry, and alignment can be regulated by the hierarchical micro/nano-structures. The results are in harmony with Subramony et al. [57]. According to the above results, it is the joint effect of hydrophobic property, the dimensional effect of structure, and reasonable Ag^+^ release rate that leads to the antibacterial activity and good cytocompatibility.

## Conclusions

In summary, the combined GA-BP neural network is successfully developed for predicting the laser induced surface structures, which is used for regulating the surface micro/nano-structures. The prepared samples show hydrophobicity, which can prevent the burst release of Ag^+^ in the initial stage and make the Ag^+^ release rate meet the clinical requirements. The AgNPs modified hierarchical micro/nano-structured surface shows both good cytocompatibility toward MC3T3-E1 cells and good antibacterial effects against *E. coli* and *S. aureus* bacteria. The bacteria behavior is inhibited by hierarchical micro/nano-structures and the bacteria is killed by AgNPs. MCs1surface is the best antibacterial surface against *E. coli* (89.27%), while the LIPSS surface is the best antibacterial surface against *S. aureus* (65.4%). The joint effect of hydrophobic property, the dimensional effect of structure, and reasonable Ag^+^ release rate lead to antibacterial activity and cytocompatibility. The reported method offers an effective strategy for guiding the design of flexible and effective antimicrobial implant surfaces.

## Supplementary Information

**Additional file 1: Table S1.** Process parameters and experiment results in GA-BP

## Data Availability

All data generated and analyzed during this research are included in this published article.
