# Supplementary material for: Artificial neural network for cytocompatibility and antibacterial enhancement induced by femtosecond laser micro/nano structures
Source: J Nanobiotechnology. 2022 Aug 6;20:365. doi: 10.1186/s12951-022-01578-4 (PMC9357338; doi:10.1186/s12951-022-01578-4)
Supplement: Supplementary file 1 — Additional file 1: Table S1. Process parameters and experiment results in GA-BP [file 12951_2022_1578_MOESM1_ESM.docx]

**Supporting Information for**

**Artificial Neural Network for Cytocompatibility and Antibacterial Enhancement Induced by Femtosecond Laser Micro/nano Structures**

**Table.S1** **Process parameters and experiment results in GA-BP**

| **No.** | **P**  **(W)** | **F**  **(KHz)** | **T** | **V**  **(mm/s)** | **PL**  **(nm)** | **WL**  **(nm)** | **DM**  **(μm)** | **PM**  **(μm)** | **No.** | **P**  **(W)** | **F**  **(KHz)** | **T** | **V**  **(mm/s)** | **PL**  **(nm)** | **WL**  **(nm)** | **DM**  **(μm)** | **PM**  **(μm)** |
| --- | --- | --- | --- | --- | --- | --- | --- | --- | --- | --- | --- | --- | --- | --- | --- | --- | --- |
| 1 | 1 | 100 | 10 | 500 | 771.21 | 803.21 | 0 | 0 | 129 | 3 | 100 | 10 | 500 | 738.62 | 776.99 | 20.12 | 21.22 |
| 2 | 1 | 100 | 10 | 800 | 700.63 | 738.52 | 0 | 0 | 130 | 3 | 100 | 10 | 800 | 661.06 | 692.98 | 17.53 | 18.29 |
| 3 | 1 | 100 | 10 | 1500 | 631.29 | 673.29 | 0 | 0 | 131 | 3 | 100 | 10 | 1500 | 609.13 | 644.22 | 16.70 | 17.09 |
| 4 | 1 | 100 | 10 | 2000 | 602.19 | 624.19 | 0 | 0 | 132 | 3 | 100 | 10 | 2000 | 574.51 | 586.84 | 14.77 | 16.38 |
| 5 | 1 | 100 | 30 | 500 | 786.27 | 816.28 | 0 | 0 | 133 | 3 | 100 | 30 | 500 | 749.65 | 774.60 | 18.50 | 19.46 |
| 6 | 1 | 100 | 30 | 800 | 748.08 | 776.08 | 0 | 0 | 134 | 3 | 100 | 30 | 800 | 714.21 | 744.44 | 16.80 | 18.54 |
| 7 | 1 | 100 | 30 | 1500 | 728.33 | 774.33 | 0 | 0 | 135 | 3 | 100 | 30 | 1500 | 693.54 | 739.51 | 13.18 | 15.15 |
| 8 | 1 | 100 | 30 | 2000 | 692.97 | 750.97 | 0 | 0 | 136 | 3 | 100 | 30 | 2000 | 657.09 | 703.98 | 11.33 | 13.25 |
| 9 | 1 | 100 | 50 | 500 | 850.78 | 883.78 | 0 | 0 | 137 | 3 | 100 | 50 | 500 | 801.61 | 839.29 | 16.59 | 18.78 |
| 10 | 1 | 100 | 50 | 800 | 836.95 | 879.95 | 0 | 0 | 138 | 3 | 100 | 50 | 800 | 789.04 | 849.24 | 15.35 | 15.67 |
| 11 | 1 | 100 | 50 | 1500 | 796.48 | 844.48 | 0 | 0 | 139 | 3 | 100 | 50 | 1500 | 771.62 | 814.85 | 10.58 | 13.81 |
| 12 | 1 | 100 | 50 | 2000 | 724.24 | 782.24 | 0 | 0 | 140 | 3 | 100 | 50 | 2000 | 677.40 | 739.30 | 9.25 | 11.33 |
| 13 | 1 | 100 | 70 | 500 | 896.58 | 934.55 | 0 | 0 | 141 | 3 | 100 | 70 | 500 | 869.62 | 897.08 | 14.72 | 16.85 |
| 14 | 1 | 100 | 70 | 800 | 843.3 | 875.39 | 0 | 0 | 142 | 3 | 100 | 70 | 800 | 798.35 | 845.06 | 12.82 | 13.90 |
| 15 | 1 | 100 | 70 | 1500 | 798.58 | 851.58 | 0 | 0 | 143 | 3 | 100 | 70 | 1500 | 757.84 | 826.06 | 12.48 | 13.75 |
| 16 | 1 | 100 | 70 | 2000 | 767.94 | 792.94 | 0 | 0 | 144 | 3 | 100 | 70 | 2000 | 721.52 | 745.73 | 8.53 | 11.01 |
| 17 | 1 | 200 | 10 | 500 | 696.21 | 718.21 | 0 | 0 | 145 | 3 | 200 | 10 | 500 | 666.24 | 678.70 | 13.14 | 12.56 |
| 18 | 1 | 200 | 10 | 800 | 677.09 | 729.09 | 0 | 0 | 146 | 3 | 200 | 10 | 800 | 653.72 | 685.77 | 11.61 | 10.88 |
| 19 | 1 | 200 | 10 | 1500 | 657.43 | 691.43 | 0 | 0 | 147 | 3 | 200 | 10 | 1500 | 633.05 | 651.72 | 8.19 | 8.77 |
| 20 | 1 | 200 | 10 | 2000 | 633.52 | 677.52 | 0 | 0 | 148 | 3 | 200 | 10 | 2000 | 587.89 | 651.77 | 6.37 | 6.88 |
| 21 | 1 | 200 | 30 | 500 | 742.89 | 769.89 | 0 | 0 | 149 | 3 | 200 | 30 | 500 | 703.49 | 750.74 | 11.21 | 11.94 |
| 22 | 1 | 200 | 30 | 800 | 719.64 | 767.64 | 0 | 0 | 150 | 3 | 200 | 30 | 800 | 693.18 | 742.70 | 8.39 | 10.49 |
| 23 | 1 | 200 | 30 | 1500 | 694.58 | 741.58 | 0 | 0 | 151 | 3 | 200 | 30 | 1500 | 653.65 | 711.68 | 8.25 | 8.85 |
| 24 | 1 | 200 | 30 | 2000 | 653.5 | 693.54 | 0 | 0 | 152 | 3 | 200 | 30 | 2000 | 633.30 | 674.43 | 6.21 | 7.20 |
| 25 | 1 | 200 | 50 | 500 | 800.53 | 847.53 | 0 | 0 | 153 | 3 | 200 | 50 | 500 | 776.77 | 825.99 | 9.97 | 11.47 |
| 26 | 1 | 200 | 50 | 800 | 756.68 | 779.68 | 0 | 0 | 154 | 3 | 200 | 50 | 800 | 714.45 | 760.40 | 8.74 | 10.74 |
| 27 | 1 | 200 | 50 | 1500 | 713.95 | 744.95 | 0 | 0 | 155 | 3 | 200 | 50 | 1500 | 685.44 | 702.62 | 6.91 | 7.48 |
| 28 | 1 | 200 | 50 | 2000 | 698.72 | 732.72 | 0 | 0 | 156 | 3 | 200 | 50 | 2000 | 678.05 | 695.60 | 4.71 | 6.01 |
| 29 | 1 | 200 | 70 | 500 | 824.06 | 861.06 | 0 | 0 | 157 | 3 | 200 | 70 | 500 | 782.52 | 838.16 | 9.08 | 9.46 |
| 30 | 1 | 200 | 70 | 800 | 795.02 | 829.02 | 0 | 0 | 158 | 3 | 200 | 70 | 800 | 759.10 | 789.19 | 8.08 | 9.44 |
| 31 | 1 | 200 | 70 | 1500 | 752.15 | 779.15 | 0 | 0 | 159 | 3 | 200 | 70 | 1500 | 730.95 | 740.34 | 6.54 | 8.23 |
| 32 | 1 | 200 | 70 | 2000 | 720.35 | 759.35 | 0 | 0 | 160 | 3 | 200 | 70 | 2000 | 692.01 | 719.82 | 3.17 | 4.09 |
| 33 | 1 | 300 | 10 | 500 | 680.32 | 703.32 | 0 | 0 | 161 | 3 | 300 | 10 | 500 | 657.96 | 653.40 | 0 | 0 |
| 34 | 1 | 300 | 10 | 800 | 653.02 | 711.02 | 0 | 0 | 162 | 3 | 300 | 10 | 800 | 623.99 | 686.07 | 0 | 0 |
| 35 | 1 | 300 | 10 | 1500 | 619.14 | 641.14 | 0 | 0 | 163 | 3 | 300 | 10 | 1500 | 598.35 | 616.24 | 0 | 0 |
| 36 | 1 | 300 | 10 | 2000 | 602.36 | 643.36 | 0 | 0 | 164 | 3 | 300 | 10 | 2000 | 575.63 | 619.13 | 0 | 0 |
| 37 | 1 | 300 | 30 | 500 | 712.33 | 763.33 | 0 | 0 | 165 | 3 | 300 | 30 | 500 | 687.80 | 727.61 | 0 | 0 |
| 38 | 1 | 300 | 30 | 800 | 694.99 | 742.99 | 0 | 0 | 166 | 3 | 300 | 30 | 800 | 673.63 | 719.62 | 0 | 0 |
| 39 | 1 | 300 | 30 | 1500 | 658.85 | 715.85 | 0 | 0 | 167 | 3 | 300 | 30 | 1500 | 629.08 | 695.57 | 0 | 0 |
| 40 | 1 | 300 | 30 | 2000 | 619.79 | 670.79 | 0 | 0 | 168 | 3 | 300 | 30 | 2000 | 589.77 | 642.90 | 0 | 0 |
| 41 | 1 | 300 | 50 | 500 | 786.55 | 834.55 | 0 | 0 | 169 | 3 | 300 | 50 | 500 | 737.21 | 815.39 | 0 | 0 |
| 42 | 1 | 300 | 50 | 800 | 737.46 | 771.46 | 0 | 0 | 170 | 3 | 300 | 50 | 800 | 701.61 | 745.51 | 0 | 0 |
| 43 | 1 | 300 | 50 | 1500 | 719.01 | 771.01 | 0 | 0 | 171 | 3 | 300 | 50 | 1500 | 673.91 | 729.47 | 0 | 0 |
| 44 | 1 | 300 | 50 | 2000 | 699.26 | 723.26 | 0 | 0 | 172 | 3 | 300 | 50 | 2000 | 654.92 | 676.89 | 0 | 0 |
| 45 | 1 | 300 | 70 | 500 | 798.28 | 838.28 | 0 | 0 | 173 | 3 | 300 | 70 | 500 | 772.17 | 819.27 | 0 | 0 |
| 46 | 1 | 300 | 70 | 800 | 762.04 | 813.04 | 0 | 0 | 174 | 3 | 300 | 70 | 800 | 720.57 | 790.36 | 0 | 0 |
| 47 | 1 | 300 | 70 | 1500 | 735.35 | 758.35 | 0 | 0 | 175 | 3 | 300 | 70 | 1500 | 695.97 | 721.21 | 0 | 0 |
| 48 | 1 | 300 | 70 | 2000 | 716.19 | 748.19 | 0 | 0 | 176 | 3 | 300 | 70 | 2000 | 686.53 | 718.43 | 0 | 0 |
| 49 | 1 | 400 | 10 | 500 | 823.92 | 873.92 | 0 | 0 | 177 | 3 | 400 | 10 | 500 | 782.44 | 854.03 | 0 | 0 |
| 50 | 1 | 400 | 10 | 800 | 806.73 | 835.73 | 0 | 0 | 178 | 3 | 400 | 10 | 800 | 771.40 | 791.17 | 0 | 0 |
| 51 | 1 | 400 | 10 | 1500 | 792.61 | 834.61 | 0 | 0 | 179 | 3 | 400 | 10 | 1500 | 762.42 | 786.25 | 0 | 0 |
| 52 | 1 | 400 | 10 | 2000 | 768.79 | 788.79 | 0 | 0 | 180 | 3 | 400 | 10 | 2000 | 741.85 | 768.74 | 0 | 0 |
| 53 | 1 | 400 | 30 | 500 | 883.91 | 904.91 | 0 | 0 | 181 | 3 | 400 | 30 | 500 | 836.94 | 863.73 | 0 | 0 |
| 54 | 1 | 400 | 30 | 800 | 863.54 | 915.87 | 0 | 0 | 182 | 3 | 400 | 30 | 800 | 822.16 | 890.37 | 0 | 0 |
| 55 | 1 | 400 | 30 | 1500 | 814.27 | 859.27 | 0 | 0 | 183 | 3 | 400 | 30 | 1500 | 785.40 | 827.05 | 0 | 0 |
| 56 | 1 | 400 | 30 | 2000 | 790.14 | 821.14 | 0 | 0 | 184 | 3 | 400 | 30 | 2000 | 766.19 | 785.19 | 0 | 0 |
| 57 | 1 | 400 | 50 | 500 | 899.02 | 956.02 | 0 | 0 | 185 | 3 | 400 | 50 | 500 | 858.12 | 936.51 | 0 | 0 |
| 58 | 1 | 400 | 50 | 800 | 874.17 | 913.17 | 0 | 0 | 186 | 3 | 400 | 50 | 800 | 829.83 | 877.12 | 0 | 0 |
| 59 | 1 | 400 | 50 | 1500 | 852.22 | 909.22 | 0 | 0 | 187 | 3 | 400 | 50 | 1500 | 821.68 | 876.18 | 0 | 0 |
| 60 | 1 | 400 | 50 | 2000 | 809.96 | 863.96 | 0 | 0 | 188 | 3 | 400 | 50 | 2000 | 783.03 | 827.95 | 0 | 0 |
| 61 | 1 | 400 | 70 | 500 | 923.79 | 949.77 | 0 | 0 | 189 | 3 | 400 | 70 | 500 | 895.81 | 905.53 | 0 | 0 |
| 62 | 1 | 400 | 70 | 800 | 887.78 | 929.78 | 0 | 0 | 190 | 3 | 400 | 70 | 800 | 851.15 | 881.43 | 0 | 0 |
| 63 | 1 | 400 | 70 | 1500 | 862.58 | 888.58 | 0 | 0 | 191 | 3 | 400 | 70 | 1500 | 818.84 | 842.35 | 0 | 0 |
| 64 | 1 | 400 | 70 | 2000 | 846.71 | 871.71 | 0 | 0 | 192 | 3 | 400 | 70 | 2000 | 808.92 | 851.89 | 0 | 0 |
| 65 | 2 | 100 | 10 | 500 | 789.65 | 813.65 | 12.85 | 15.47 | 193 | 4 | 100 | 10 | 500 | 763.44 | 781.74 | 24.12 | 25.64 |
| 66 | 2 | 100 | 10 | 800 | 715.94 | 773.39 | 10.14 | 12.02 | 194 | 4 | 100 | 10 | 800 | 694.62 | 733.28 | 18.11 | 20.80 |
| 67 | 2 | 100 | 10 | 1500 | 646.01 | 669.01 | 9.57 | 10.28 | 195 | 4 | 100 | 10 | 1500 | 598.85 | 646.58 | 18.66 | 20.40 |
| 68 | 2 | 100 | 10 | 2000 | 614.57 | 652.57 | 5.28 | 8.15 | 196 | 4 | 100 | 10 | 2000 | 569.63 | 628.58 | 17.09 | 17.32 |
| 69 | 2 | 100 | 30 | 500 | 804.28 | 851.28 | 11.53 | 14.89 | 197 | 4 | 100 | 30 | 500 | 782.96 | 805.41 | 19.76 | 20.55 |
| 70 | 2 | 100 | 30 | 800 | 764.47 | 818.45 | 9.69 | 13.74 | 198 | 4 | 100 | 30 | 800 | 743.59 | 768.53 | 19.37 | 22.24 |
| 71 | 2 | 100 | 30 | 1500 | 745.38 | 778.63 | 6.93 | 10.91 | 199 | 4 | 100 | 30 | 1500 | 703.62 | 736.97 | 18.56 | 20.56 |
| 72 | 2 | 100 | 30 | 2000 | 703.33 | 724.35 | 4.61 | 8.6 | 200 | 4 | 100 | 30 | 2000 | 675.73 | 698.77 | 16.61 | 17.05 |
| 73 | 2 | 100 | 50 | 500 | 865.95 | 915.95 | 9.56 | 10.65 | 201 | 4 | 100 | 50 | 500 | 838.64 | 883.22 | 22.01 | 23.48 |
| 74 | 2 | 100 | 50 | 800 | 854.32 | 904.33 | 8.26 | 8.02 | 202 | 4 | 100 | 50 | 800 | 824.63 | 868.80 | 19.12 | 16.25 |
| 75 | 2 | 100 | 50 | 1500 | 814.68 | 849.68 | 6.33 | 7.42 | 203 | 4 | 100 | 50 | 1500 | 783.38 | 818.99 | 15.29 | 14.35 |
| 76 | 2 | 100 | 50 | 2000 | 734.55 | 774.53 | 4.22 | 6.46 | 204 | 4 | 100 | 50 | 2000 | 701.65 | 745.67 | 10.60 | 12.09 |
| 77 | 2 | 100 | 70 | 500 | 906.64 | 929.64 | 8.28 | 10.57 | 205 | 4 | 100 | 70 | 500 | 863.45 | 893.85 | 15.87 | 21.28 |
| 78 | 2 | 100 | 70 | 800 | 855.41 | 884.41 | 6.67 | 8.3 | 206 | 4 | 100 | 70 | 800 | 821.08 | 864.73 | 14.57 | 17.89 |
| 79 | 2 | 100 | 70 | 1500 | 812.75 | 846.75 | 5.22 | 5.11 | 207 | 4 | 100 | 70 | 1500 | 763.29 | 824.77 | 13.87 | 17.74 |
| 80 | 2 | 100 | 70 | 2000 | 787.14 | 822.14 | 2.29 | 4.06 | 208 | 4 | 100 | 70 | 2000 | 745.11 | 792.37 | 11.12 | 13.96 |
| 81 | 2 | 200 | 10 | 500 | 713.86 | 754.67 | 0 | 0 | 209 | 4 | 200 | 10 | 500 | 693.15 | 722.00 | 16.78 | 15.53 |
| 82 | 2 | 200 | 10 | 800 | 690.81 | 724.81 | 0 | 0 | 210 | 4 | 200 | 10 | 800 | 651.15 | 686.65 | 15.97 | 14.76 |
| 83 | 2 | 200 | 10 | 1500 | 676.29 | 732.29 | 0 | 0 | 211 | 4 | 200 | 10 | 1500 | 627.09 | 691.58 | 12.87 | 10.61 |
| 84 | 2 | 200 | 10 | 2000 | 644.85 | 698.83 | 0 | 0 | 212 | 4 | 200 | 10 | 2000 | 606.03 | 669.04 | 8.10 | 11.16 |
| 85 | 2 | 200 | 30 | 500 | 752.27 | 783.27 | 0 | 0 | 213 | 4 | 200 | 30 | 500 | 704.39 | 760.56 | 13.29 | 15.54 |
| 86 | 2 | 200 | 30 | 800 | 740.38 | 800.38 | 0 | 0 | 214 | 4 | 200 | 30 | 800 | 692.10 | 780.79 | 11.34 | 12.71 |
| 87 | 2 | 200 | 30 | 1500 | 713.12 | 763.12 | 0 | 0 | 215 | 4 | 200 | 30 | 1500 | 667.83 | 737.58 | 13.01 | 9.83 |
| 88 | 2 | 200 | 30 | 2000 | 664.91 | 722.91 | 0 | 0 | 216 | 4 | 200 | 30 | 2000 | 619.10 | 695.96 | 9.52 | 10.36 |
| 89 | 2 | 200 | 50 | 500 | 817.84 | 840.84 | 0 | 0 | 217 | 4 | 200 | 50 | 500 | 797.13 | 814.77 | 11.98 | 14.20 |
| 90 | 2 | 200 | 50 | 800 | 767.34 | 805.34 | 0 | 0 | 218 | 4 | 200 | 50 | 800 | 729.92 | 766.33 | 11.13 | 12.31 |
| 91 | 2 | 200 | 50 | 1500 | 725.75 | 768.75 | 0 | 0 | 219 | 4 | 200 | 50 | 1500 | 696.07 | 736.39 | 12.21 | 13.11 |
| 92 | 2 | 200 | 50 | 2000 | 718.13 | 762.13 | 0 | 0 | 220 | 4 | 200 | 50 | 2000 | 695.50 | 712.52 | 9.70 | 10.65 |
| 93 | 2 | 200 | 70 | 500 | 835.51 | 887.51 | 0 | 0 | 221 | 4 | 200 | 70 | 500 | 790.52 | 850.98 | 14.62 | 12.05 |
| 94 | 2 | 200 | 70 | 800 | 809.89 | 831.89 | 0 | 0 | 222 | 4 | 200 | 70 | 800 | 789.03 | 804.05 | 12.63 | 13.64 |
| 95 | 2 | 200 | 70 | 1500 | 763.48 | 811.74 | 0 | 0 | 223 | 4 | 200 | 70 | 1500 | 743.22 | 790.40 | 7.40 | 10.00 |
| 96 | 2 | 200 | 70 | 2000 | 740.42 | 771.42 | 0 | 0 | 224 | 4 | 200 | 70 | 2000 | 713.76 | 751.92 | 3.62 | 6.60 |
| 97 | 2 | 300 | 10 | 500 | 698.79 | 721.79 | 0 | 0 | 225 | 4 | 300 | 10 | 500 | 672.71 | 687.58 | 0 | 0 |
| 98 | 2 | 300 | 10 | 800 | 666.05 | 712.74 | 0 | 0 | 226 | 4 | 300 | 10 | 800 | 635.81 | 668.54 | 0 | 0 |
| 99 | 2 | 300 | 10 | 1500 | 637.76 | 684.76 | 0 | 0 | 227 | 4 | 300 | 10 | 1500 | 617.49 | 635.94 | 0 | 0 |
| 100 | 2 | 300 | 10 | 2000 | 621.33 | 665.33 | 0 | 0 | 228 | 4 | 300 | 10 | 2000 | 582.08 | 618.74 | 0 | 0 |
| 101 | 2 | 300 | 30 | 500 | 724.34 | 771.34 | 0 | 0 | 229 | 4 | 300 | 30 | 500 | 678.10 | 730.14 | 0 | 0 |
| 102 | 2 | 300 | 30 | 800 | 711.84 | 770.84 | 0 | 0 | 230 | 4 | 300 | 30 | 800 | 666.12 | 740.29 | 0 | 0 |
| 103 | 2 | 300 | 30 | 1500 | 674.93 | 717.93 | 0 | 0 | 231 | 4 | 300 | 30 | 1500 | 638.78 | 683.26 | 0 | 0 |
| 104 | 2 | 300 | 30 | 2000 | 630.97 | 666.97 | 0 | 0 | 232 | 4 | 300 | 30 | 2000 | 581.11 | 645.94 | 0 | 0 |
| 105 | 2 | 300 | 50 | 500 | 801.86 | 842.86 | 0 | 0 | 233 | 4 | 300 | 50 | 500 | 780.62 | 799.89 | 0 | 0 |
| 106 | 2 | 300 | 50 | 800 | 749.67 | 804.67 | 0 | 0 | 234 | 4 | 300 | 50 | 800 | 717.34 | 774.46 | 0 | 0 |
| 107 | 2 | 300 | 50 | 1500 | 733.85 | 792.85 | 0 | 0 | 235 | 4 | 300 | 50 | 1500 | 695.47 | 743.33 | 0 | 0 |
| 108 | 2 | 300 | 50 | 2000 | 719.37 | 779.37 | 0 | 0 | 236 | 4 | 300 | 50 | 2000 | 689.08 | 748.25 | 0 | 0 |
| 109 | 2 | 300 | 70 | 500 | 818.44 | 839.44 | 0 | 0 | 237 | 4 | 300 | 70 | 500 | 784.41 | 803.55 | 0 | 0 |
| 110 | 2 | 300 | 70 | 800 | 782.06 | 833.06 | 0 | 0 | 238 | 4 | 300 | 70 | 800 | 745.99 | 813.55 | 0 | 0 |
| 111 | 2 | 300 | 70 | 1500 | 745.98 | 766.98 | 0 | 0 | 239 | 4 | 300 | 70 | 1500 | 724.53 | 725.89 | 0 | 0 |
| 112 | 2 | 300 | 70 | 2000 | 730.55 | 777.55 | 0 | 0 | 240 | 4 | 300 | 70 | 2000 | 700.56 | 757.32 | 0 | 0 |
| 113 | 2 | 400 | 10 | 500 | 838.21 | 888.21 | 0 | 0 | 241 | 4 | 400 | 10 | 500 | 803.60 | 847.11 | 0 | 0 |
| 114 | 2 | 400 | 10 | 800 | 818.32 | 844.32 | 0 | 0 | 242 | 4 | 400 | 10 | 800 | 769.30 | 798.11 | 0 | 0 |
| 115 | 2 | 400 | 10 | 1500 | 810.55 | 868.55 | 0 | 0 | 243 | 4 | 400 | 10 | 1500 | 778.14 | 839.83 | 0 | 0 |
| 116 | 2 | 400 | 10 | 2000 | 780.49 | 818.49 | 0 | 0 | 244 | 4 | 400 | 10 | 2000 | 738.03 | 788.00 | 0 | 0 |
| 117 | 2 | 400 | 30 | 500 | 893.84 | 941.84 | 0 | 0 | 245 | 4 | 400 | 30 | 500 | 871.99 | 909.90 | 0 | 0 |
| 118 | 2 | 400 | 30 | 800 | 874.93 | 899.93 | 0 | 0 | 246 | 4 | 400 | 30 | 800 | 842.47 | 879.53 | 0 | 0 |
| 119 | 2 | 400 | 30 | 1500 | 828.51 | 851.51 | 0 | 0 | 247 | 4 | 400 | 30 | 1500 | 793.59 | 826.31 | 0 | 0 |
| 120 | 2 | 400 | 30 | 2000 | 810.95 | 836.95 | 0 | 0 | 248 | 4 | 400 | 30 | 2000 | 787.43 | 803.70 | 0 | 0 |
| 121 | 2 | 400 | 50 | 500 | 916.38 | 939.38 | 0 | 0 | 249 | 4 | 400 | 50 | 500 | 891.62 | 898.84 | 0 | 0 |
| 122 | 2 | 400 | 50 | 800 | 884.8 | 925.8 | 0 | 0 | 250 | 4 | 400 | 50 | 800 | 865.63 | 896.91 | 0 | 0 |
| 123 | 2 | 400 | 50 | 1500 | 867.09 | 923.09 | 0 | 0 | 251 | 4 | 400 | 50 | 1500 | 823.23 | 893.29 | 0 | 0 |
| 124 | 2 | 400 | 50 | 2000 | 822.2 | 878.2 | 0 | 0 | 252 | 4 | 400 | 50 | 2000 | 785.38 | 847.73 | 0 | 0 |
| 125 | 2 | 400 | 70 | 500 | 938.7 | 968.7 | 0 | 0 | 253 | 4 | 400 | 70 | 500 | 919.08 | 938.13 | 0 | 0 |
| 126 | 2 | 400 | 70 | 800 | 907.54 | 948.54 | 0 | 0 | 254 | 4 | 400 | 70 | 800 | 880.09 | 904.15 | 0 | 0 |
| 127 | 2 | 400 | 70 | 1500 | 878.4 | 904.4 | 0 | 0 | 255 | 4 | 400 | 70 | 1500 | 839.08 | 859.12 | 0 | 0 |
| 128 | 2 | 400 | 70 | 2000 | 860.52 | 887.52 | 0 | 0 | 256 | 4 | 400 | 70 | 2000 | 822.79 | 864.09 | 0 | 0 |

0
